# Environmental genome-wide association studies across precipitation regimes reveal that the E3 ubiquitin ligase MBR1 regulates plant adaptation to rainy environments

**DOI:** 10.1016/j.xplc.2024.101074

**Published:** 2024-08-31

**Authors:** Simone Castellana, Paolo Maria Triozzi, Matteo Dell’Acqua, Elena Loreti, Pierdomenico Perata

**Affiliations:** 1 Institute of Plant Sciences, Scuola Superiore Sant’Anna, Via Guidiccioni 10, San Giuliano Terme (Pisa), Italy; 2 Institute of Agricultural Biology and Biotechnology, CNR, National Research Council, Via Moruzzi 1, Pisa, Italy

**Keywords:** eGWAS, hypoxia, *Arabidopsis thaliana*, abiotic stress, natural variation, *MBR1*

## Abstract

In an era characterized by rapidly changing and less-predictable weather conditions fueled by the climate crisis, understanding the mechanisms underlying local adaptation in plants is of paramount importance for the conservation of species. As the frequency and intensity of extreme precipitation events increase, so are the flooding events resulting from soil water saturation. The subsequent onset of hypoxic stress is one of the leading causes of crop damage and yield loss. By combining genomics and remote sensing data, it is now possible to probe natural plant populations that have evolved in different rainfall regimes and look for molecular adaptation to hypoxia. Here, using an environmental genome-wide association study (eGWAS) of 934 non-redundant georeferenced *Arabidopsis* ecotypes, we have identified functional variants of the gene *MED25 BINDING RING-H2 PROTEIN 1* (*MBR1*). This gene encodes a ubiquitin-protein ligase that regulates MEDIATOR25 (MED25), part of a multiprotein complex that interacts with transcription factors that act as key drivers of the hypoxic response in *Arabidopsis*, namely the RELATED TO AP2 proteins RAP2.2 and RAP2.12. Through experimental validation, we show that natural variants of *MBR1* have different effects on the stability of MED25 and, in turn, on hypoxia tolerance. This study also highlights the pivotal role of the MBR1/MED25 module in establishing a comprehensive hypoxic response. Our findings show that molecular candidates for plant environmental adaptation can be effectively mined from large datasets. This thus supports the need for integration of forward and reverse genetics with robust molecular physiology validation of outcomes.

## Teaser

Extreme precipitation events lead to flooding and hypoxia, influencing plant survival. This study reports an investigation of molecular adaptations to hypoxia by combining genomics and climatic data in a natural population of 934 non-redundant georeferenced *Arabidopsis* ecotypes that have evolved in different rainfall regimes. It reveals that MED25 BINDING RING-H2 PROTEIN 1 is a key driver of the hypoxic response and survival in *Arabidopsis* growing in rainy environments.

## Introduction

Local adaptation in plant populations results from a combination of evolutionary history and phenotypic plasticity (Williams 1966; Kawecki and Ebert, 2004). Combinations of phenotypes that favor adaptation, resulting in increased fitness, ultimately depend on variations at the DNA level that are inherited and exchanged in the allele pool of natural populations (Leimu and Fischer, 2008). Understanding the genetic factors that confer local adaptation in an era characterized by rapidly shifting climate scenarios would enable plant biology to provide tools for the conservation and sustainable exploitation of biodiversity (Thomas et al., 2004; Urban, 2015). The rise in global temperatures has an impact on the hydrological cycle (Rind et al., 1992; Kundzewicz, 2008). If, on the one hand, heat waves and droughts are becoming increasingly common (Cook et al., 2018; Mukherjee et al., 2018), then, on the other hand, there has also been an intensification of extreme precipitation events, which have led to a higher occurrence of floods (Hirabayashi et al., 2013; Tabari, 2020). Floods are among the most severe climate catastrophes in agriculture, with an estimated impact of $21 billion for the decade 2008–2018 (FAO, 2021). Understanding the molecular basis of tolerance to abiotic stresses, including flooding, would break new ground in the development of new stress-resistant crop varieties (Forester et al., 2022).

During flood events, as heavy rainfall saturates the soil, plants may be partially submerged (a condition known as waterlogging) or completely covered by water (a state known as submergence). These conditions inhibit the gas exchange that typically occurs between the plant and the surrounding environment (Blom and Voesenek, 1996; Jackson and Colmer, 2005; Bailey-Serres and Voesenek, 2008; Loreti et al., 2016). Because oxygen (O_2_) is a critical element for energy production and other vital metabolic processes, the hypoxic conditions that arise in flooded soils can severely impact plant growth and development (Visser et al., 2003; Pucciariello et al., 2014). Plants have therefore evolved sophisticated mechanisms to compensate for transient periods of oxygen deprivation. The primary strategy is to switch from oxidative phosphorylation, which requires oxygen, to anaerobic metabolism to generate the required energy (Loreti and Perata, 2020). This process involves a finely tuned oxygen-dependent modulation of gene expression, in which one of the key pathways is the ethylene signaling pathway mediated by ERF-VII transcription factors (TFs) (GROUP VII ETHYLENE RESPONSE FACTORS) (Loreti and Perata, 2023). Under aerobic conditions, ERF-VIIs are constitutively oxidized by a class of plant enzymes called PLANT CYSTEINE OXIDASES (PCOs), which target the ERF-VII TFs for degradation via the proteasome (Weits et al., 2014; White et al., 2017), following the Cys branch of the N-degron pathway (Gibbs et al., 2011; Licausi et al., 2011). Under hypoxia, ERF-VIIs escape degradation and are stabilized, enabling transcription of the core genes necessary for plant survival (Mustroph et al., 2010).

Despite our comprehensive understanding of oxygen-sensing mechanisms, forward genetic approaches such as genome-wide association studies (GWASs) can still be used to reveal previously undiscovered regulatory elements in the hypoxic machinery. GWASs have helped to identify new genetic variants associated with traits of interest and have complemented reverse genetic approaches focused on characterizing mutants, thus contributing significantly to our understanding of the genetic basis of plant adaptation (Brachi and Borevitz, 2011; Tibbs Cortes et al., 2021). Recently, the relationship between oxygen sensing and allelic variation was studied through GWASs. Specifically, Lou et al. (2022) investigated the differential regulation of drought and flood tolerance in *Arabidopsis* populations through allelic changes in the *cis*-elements of the TF RELATED TO APETALA 2.12 (RAP2.12). Using a population of recombinant inbred lines (RILs) created by crossing *Arabidopsis* accessions from Sichuan and Tibet, the authors demonstrated that variations in the WT box and W box *cis*-elements of the RAP2.12 promoter are responsible for the differential regulation of flood and drought tolerance. The transition from one allele to another is associated with the colonization of wet environments from arid habitats, highlighting an adaptive mechanism that diversifies regulation through non-coding alleles.

Genetic variation is the primary driver of variation in a particular trait or phenotype, but GWASs unfortunately involve a resource-intensive and time-consuming method of collecting phenotypic data or creating RILs. However, environmental factors can also have a significant impact on trait variation. In fact, the trait variations observed may not be due solely to genetic factors. Instead, some of the variation may be due to environmental effects, leading to false positive or false negative GWAS results (Korte and Farlow, 2013).

To overcome these limitations, environmental GWASs (eGWASs) have been used to identify associations between genetic diversity and pedoclimatic diversity in natural populations of plants and animals. These studies exploit the distribution of genetic diversity to infer past evolutionary processes and understand their contribution to extant variation (Rellstab et al., 2015). To date, eGWASs have been used on a wide range of different plant species, from trees (Eckert et al., 2010; Sork et al., 2010) to economically important crops (Yoder et al., 2014; Gates et al., 2019; Gibson and Moyle, 2020). This has been made possible by the increasing availability of genomic information coupled with high-resolution, open-source climatic datasets derived from remote sensing and re-analysis of climate data.

The model species *Arabidopsis thaliana*, because of its well-known genetics and wide global distribution, is an ideal candidate for eGWASs (Fournier-Level et al., 2011; Hancock et al., 2011). Different studies have successfully used the eGWAS approach on *Arabidopsis*, revealing genes adapted to a wide range of environmental conditions; these include genes linked to arid climates (Exposito-Alonso et al., 2018), TFs involved in cold acclimatization (Monroe et al., 2016), and sodium transporters that regulate salt tolerance based on proximity to the coast (Baxter et al., 2010). Although eGWAS has great potential for describing the molecular mechanisms underlying adaptation, it is often constrained by a lack of means to validate the importance of allele variation in contributing to the studied traits (Lasky et al., 2023).

On the basis of these assumptions, we carried out an eGWAS to reveal potential candidate genes linked to areas of abundant rainfall. These genetic determinants can shape the capacity of an organism to withstand the severity of rainfall-related challenges, such as waterlogging and submergence. We therefore used up-to-date databases of precipitation and soil characteristics, cross-referencing them with genetic data for a global collection of *Arabidopsis*.

We identified a strong association with SNPs present in the coding sequence of the *MED25 BINDING RING-H2 PROTEIN 1* (*MBR1*) gene. *MBR1* encodes an E3-protein ubiquitin ligase that regulates the stability of a subunit of the Mediator complex (Iñigo et al., 2012), MEDIATOR25 (MED25), which in turn acts as a bridge between TFs and RNA polymerase II, ultimately regulating gene expression (Kazan, 2017). MED25 interacts with the key TFs RAP2.2 and RAP2.12 (Ou et al., 2011; Shukla et al., 2019), which are key regulators of the hypoxic response in *Arabidopsis*. Remarkably, MED25 is recruited by these two ERF-VIIs to coordinate gene expression during hypoxia in *A. thaliana* (Schippers et al., 2024).

In this study, we demonstrate how, through a cascade of events, natural variants of *MBR1* impact the stability of MED25 and, consequently, the tolerance to hypoxic stress. We also demonstrate that the MBR1/MED25 module plays a central role in establishing a comprehensive hypoxic response. Our research underscores the efficacy of integrating eGWAS with experimental gene validation, which accelerates the identification of molecular factors that contribute to plant hypoxic adaptation. We believe that this approach opens new avenues for more effective conservation and harnessing of biodiversity aimed at strengthening resilience to the climate crisis.

## Results

### Climatic and genetic diversity in the collection

**Figure 1:**
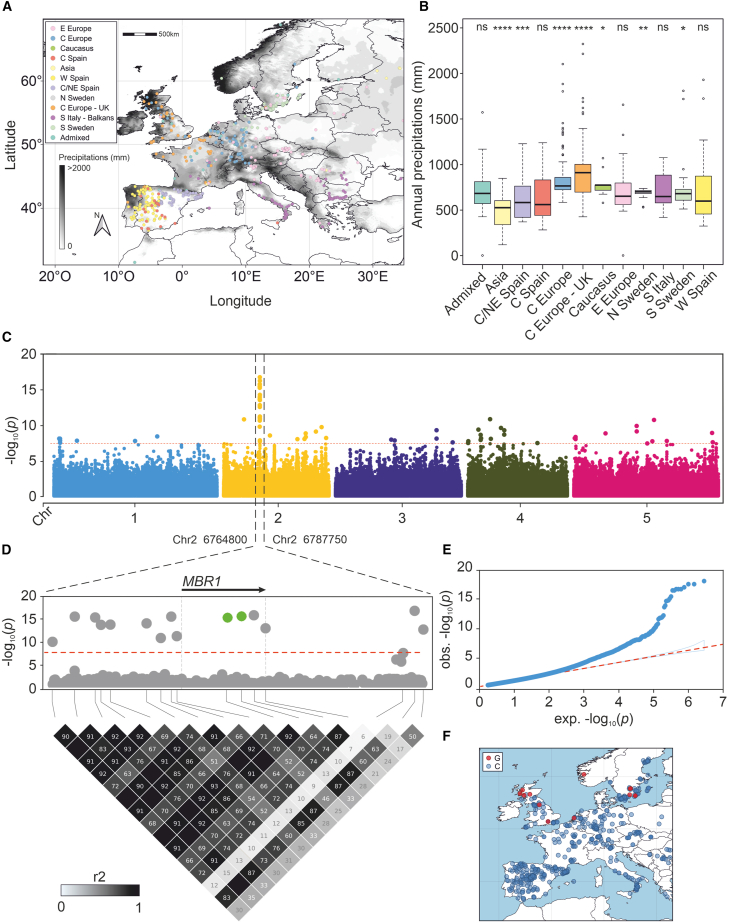
Association analysis with precipitation reveals variants in the *MBR1* gene. **(A)** Geographic locations of the 11 clusters highlighted by ADMIXTURE; the gray color scale indicates the average annual rainfall expressed in millimeters for the period 1970–2020. **(B)** Boxplot representing the distribution of accessions in relation to the annual rainfall expressed in millimeters; statistically significant differences in the mean of all samples are indicated by asterisks (Student’s *t* test; ns = non-significant; ∗*p* < 0.05; ∗∗*p* < 0.01; ∗∗∗*p* < 0.001; ∗∗∗∗*p* < 0.0001). **(C)** Manhattan plot of eGWAS for the PC1 variable; the dashed red line indicates the significance threshold set to *α* = 0.05 after Bonferroni correction. **(D)** Magnification of chromosome 2. The dashed vertical lines indicate the position of the *MBR1* gene; the green dots indicate the non-synonymous SNPs highlighted by the eGWAS analysis. Bottom, LD heatmap showing the LD patterns within the magnified region. **(E)** q-q plot showing the observed versus expected −log10(*p*) values for the GWAS for the PC1 variable. **(F)** Geographic distribution of the accessions carrying the most significant nonsynonymous SNP (SNP2 as shown in Supplemental Figure 6); the blue points represent the distribution of the reference allele (*MBR1* -SNP2- C/C), and the red points represent the distribution of the alternative allele (*MBR1wet* -SNP2- G/G).

The 1001 Genomes Project (https://1001genomes.org/index.html) provides genomic sequencing data for 1135 *Arabidopsis* accessions collected in the wild. Starting with the overall dataset, we selected 934 nonredundant georeferenced ecotypes sampled across the global growth area of *Arabidopsis* (Supplemental Figure 1A). We found that the genetic diversity present in the dataset was best summarized by 11 genetic clusters (Supplemental Table 1) in partially overlapping geographic regions (Figure 1A and Supplemental Figure 1C).

According to the WorldClim (WC) dataset, rainfall in the collection areas ranged from 118 to 2324 mm/year. Most of the accessions were sampled in areas that experienced approximately 700 mm/year (mean = 722.59 mm/year; SD = 246.57). Gridded rainfall data derived from different models may differ, so we compared the WC dataset with four other datasets, finding high consistency (Pearson’s *r* from 0.73 to 0.99; Supplemental Figure 2). We used a principal-component analysis (PCA) to summarize precipitation data from the different datasets in PC1, which explained 87.3% of the variance in rainfall data. This variable, which represented the best approximation of precipitation variation in the area of study, was used in subsequent analyses. Correlations with various soil variables were analyzed, considering the close relationship between waterlogging phenomena and soil characteristics. An analysis was performed to investigate the potential correlation between soil variables and precipitation variables, with the aim of confirming the independence of these datasets. The results demonstrated no correlation between the two sets of variables (Supplemental Figure 3). This finding confirms that the soil and precipitation variables are entirely independent and do not share overlapping information. We found that genetic clusters were characterized by different rainfall regimes (Figure 1B and Supplemental Figure 4), suggesting the possibility of local adaptation resulting from evolutionary processes.

### Environmental association analysis reveals an association with a gene located on chromosome 2

**Figure 2:**
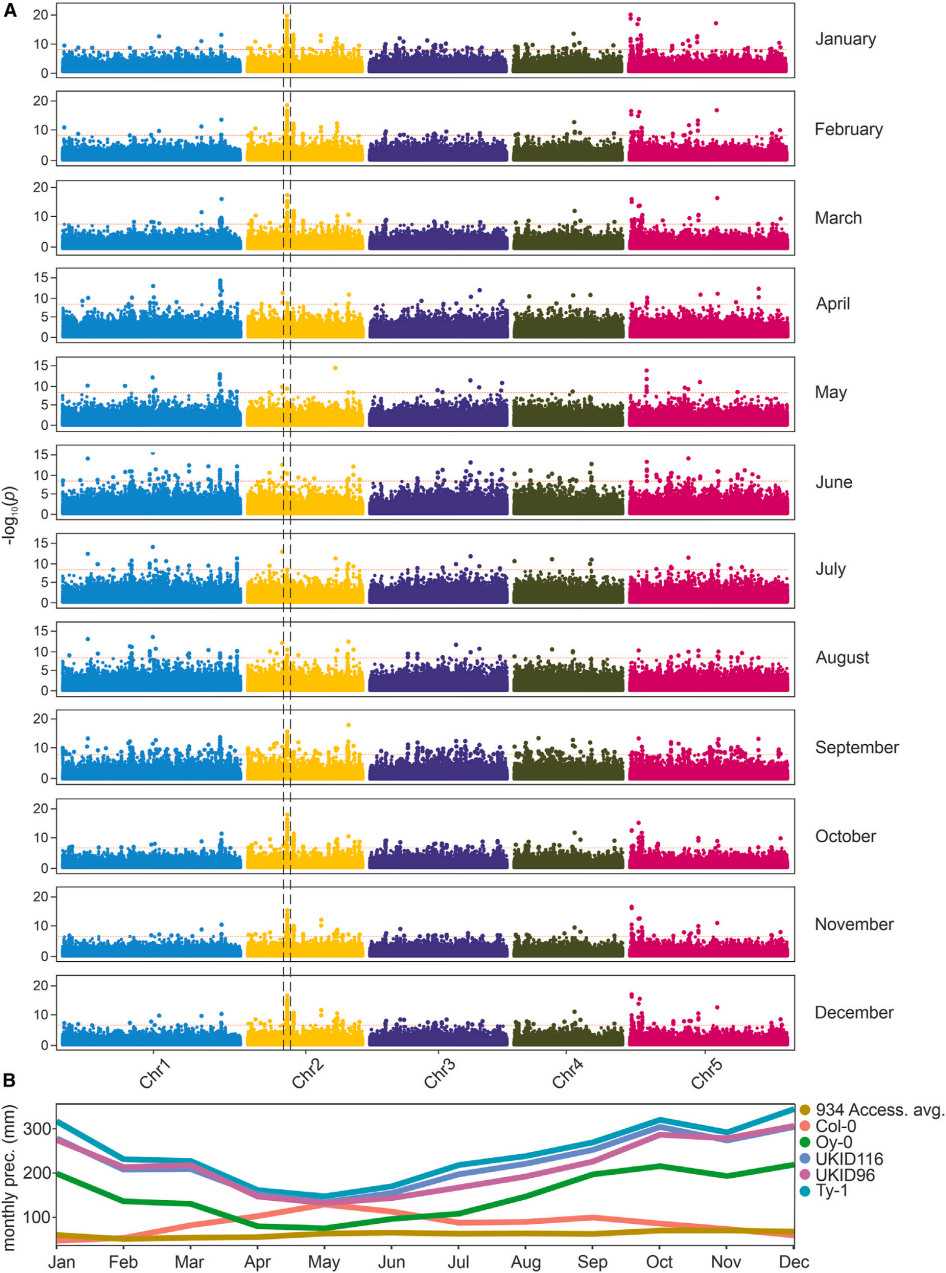
The marker-trait association tagging *MBR1* is more significant in months with higher rainfall. **(A)** Manhattan plots of the eGWAS performed with month-specific precipitation data for the period 1901–2020; dashed lines indicate the *MBR1* region as depicted in Figure 1. **(B)** Monthly precipitation values (in millimeters) associated with accessions displaying the *MBR1wet* allele at the gene locus. Rainfall values during the boreal winter are lower for Col-0 and the average of all 934 *Arabidopsis* ecotypes than for ecotypes carrying the adaptive allele.

We performed eGWASs on each individual rainfall dataset independently (Supplemental Figure 5), as well as on the derived PC1. The resulting Manhattan plot for PC1 is shown in Figure 1C. The complete list of significant associations can be found in Supplemental Table 2. To identify a set of SNPs that were most likely to contribute to differences in protein-coding genes, we focused on polymorphisms that occurred in the coding regions of genes, and specifically on missense variants. eGWASs revealed a highly significant peak located on chromosome 2, contributed by several SNPs in and around the gene *MBR1* (Figure 1D). When performing linkage disequilibrium (LD) analysis of the locus, we found that all significant SNPs belonged to the same LD block, meaning that it was not possible to resolve the association further (Figure 1D). We found two SNPs with a minor allele frequency (MAF) of 0.02 in the coding region of the *MBR1* gene model; both these SNPs were predicted to cause missense mutations and were chosen as prime candidates for further analyses. The version of the gene carried by accessions located in rainy environments that contains the two identified polymorphisms will hereafter be referred to as *MBR1wet*. The gene model with the corresponding substitutions is shown in Supplemental Figure 6. A list of accessions carrying the reference allele and the alternative allele can be found in Supplementary Table 3. We further explored environmental associations by performing the eGWAS with month-specific precipitation data, finding higher significance for the *MBR1* association from October to January, months characterized by higher rainfall values in their respective sampling locations (Figures 2A and 2B). The same SNPs located in the *MBR1* gene were obtained from an eGWAS that considered soil bulk density, a soil characteristic that is a proxy for soil water-holding capacity (Supplemental Figure 7A). This seasonal precipitation pattern aligns with the significant associations observed in the eGWAS, suggesting that the allelic variants of *MBR1* are likely adapted to environments characterized by higher winter rainfall.

### Possible involvement of MBR1 in hypoxia tolerance

**Figure 3:**
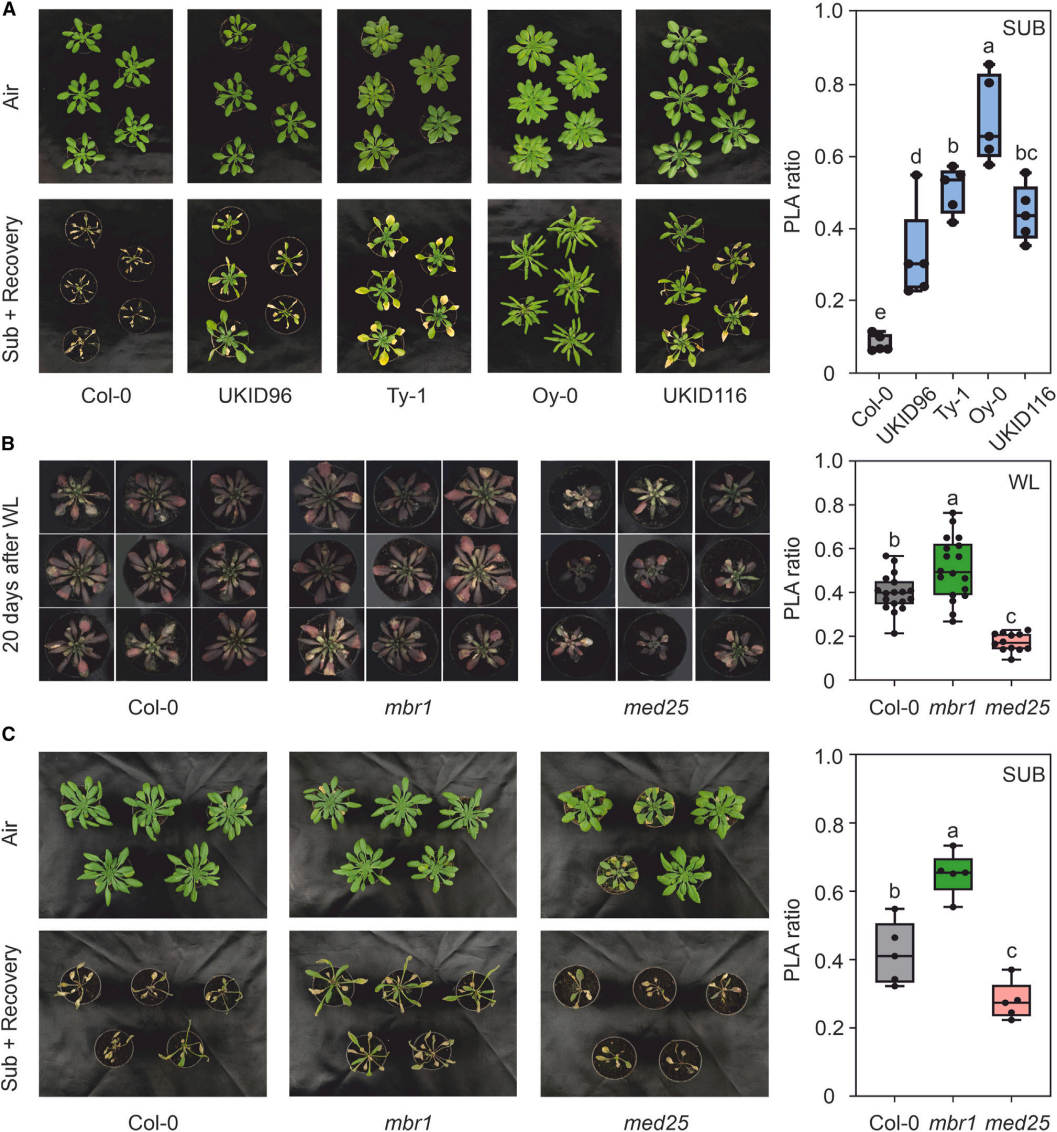
Submergence and waterlogging trials. **(A)** Effect of submergence on survival of Col-0 and accessions carrying the *MBR1wet* allele. The plants were submerged for 48 h in the dark and transferred to normal 16-h light/8-h dark conditions. Photographs were taken after 1 week of recovery. The boxplot shows the PLA ratio assessed 1 week after the end of the treatment. **(B)** Effect of waterlogging on the survival of Col-0, *mbr1*, and *med25* plants. The plants were kept in water for 20 days. Before phenotyping, the plants were randomized to help minimize potential bias and ensure that variations in environmental conditions were evenly distributed. The boxplot shows the PLA ratio assessed 20 days after the start of waterlogging. **(C)** Effect of submergence on the survival of Col-0, *mbr1*, and *med25* plants. The boxplot shows the PLA ratio assessed after 48 h of recovery following 48 h of submergence. In the boxplots, dots represent single data points, whiskers denote the minimum/maximum values, the box defines the interquartile range, the center represents the median, and the box borders represent the lower and upper quartiles. Different letters indicate differences in ANOVA tests (Tukey’s post hoc test, *p* < 0.05). The plant images were cropped and edited for clarification. The original unedited images for the waterlogging experiment can be found in Supplemental Figure 11.

*MBR1* encodes a RING-type E3 ligase that acts as a regulator of MED25, a subunit of the Mediator complex, by directing its degradation in a RING-H2-dependent manner (Iñigo et al., 2012). MED25 physically interacts with transcriptional activators, including members of the AP2/ERF family, such as two master regulators of the plant hypoxic response, RAP2.2 and RAP2.12 (Ou et al., 2011; Shukla et al., 2019). The close connection between *MBR1* and MED25, along with the interaction between MED25 and ERF-VII, led to the hypothesis of a possible link to the hypoxic response, whereby altered protein activity could affect the stability of MED25 and thus influence the plant’s response to oxygen deprivation. To test this hypothesis, we performed a submergence trial to compare the tolerance of the Col-0 genotype to that of natural ecotypes carrying the polymorphism in *MBR1*, namely UKID96, UKID116, Ty-1, and Oy-0. The results indicated that all these ecotypes demonstrated better performance compared with Col-0 (Figure 3A). However, the ability of the ecotypes to tolerate submergence better than the wild type (WT) could be related not only to mutations in the *MBR1* gene but also to a polygenic effect.

To test whether *MBR1* is directly involved in the mechanisms of hypoxia tolerance, we employed both waterlogging and submersion experiments. Initially, waterlogging was used for the Col-0, *mbr1*, and *med25* knockout genotypes to simulate partial soil flooding, a common form of mild hypoxic stress encountered in natural environments (Figure 3B). This approach enabled us to observe variations in hypoxia tolerance under conditions that mirror real-world scenarios. To ensure consistency and validate our findings, we subsequently performed submersion experiments on Col-0, *mbr1*, and *med25* genotypes (Figure 3C). The results, expressed as the plant leaf area ratio (PLA ratio) of waterlogged plants to plants kept in the air, showed that *mbr1* tolerated the stress better than Col-0 under both experimental conditions, whereas *med25* demonstrated the worst performance (Figures 3B and 3C). These results indicate that the absence of *MBR1* results in a better hypoxic response, perhaps due to the greater stability of MED25. By contrast, when MED25 is absent, the plant’s resistance to hypoxic stress is lower, providing additional evidence that MED25 plays a role in the plant hypoxic response.

### MBR1 acts as a repressor of the hypoxic transcriptional response

**Figure 4:**
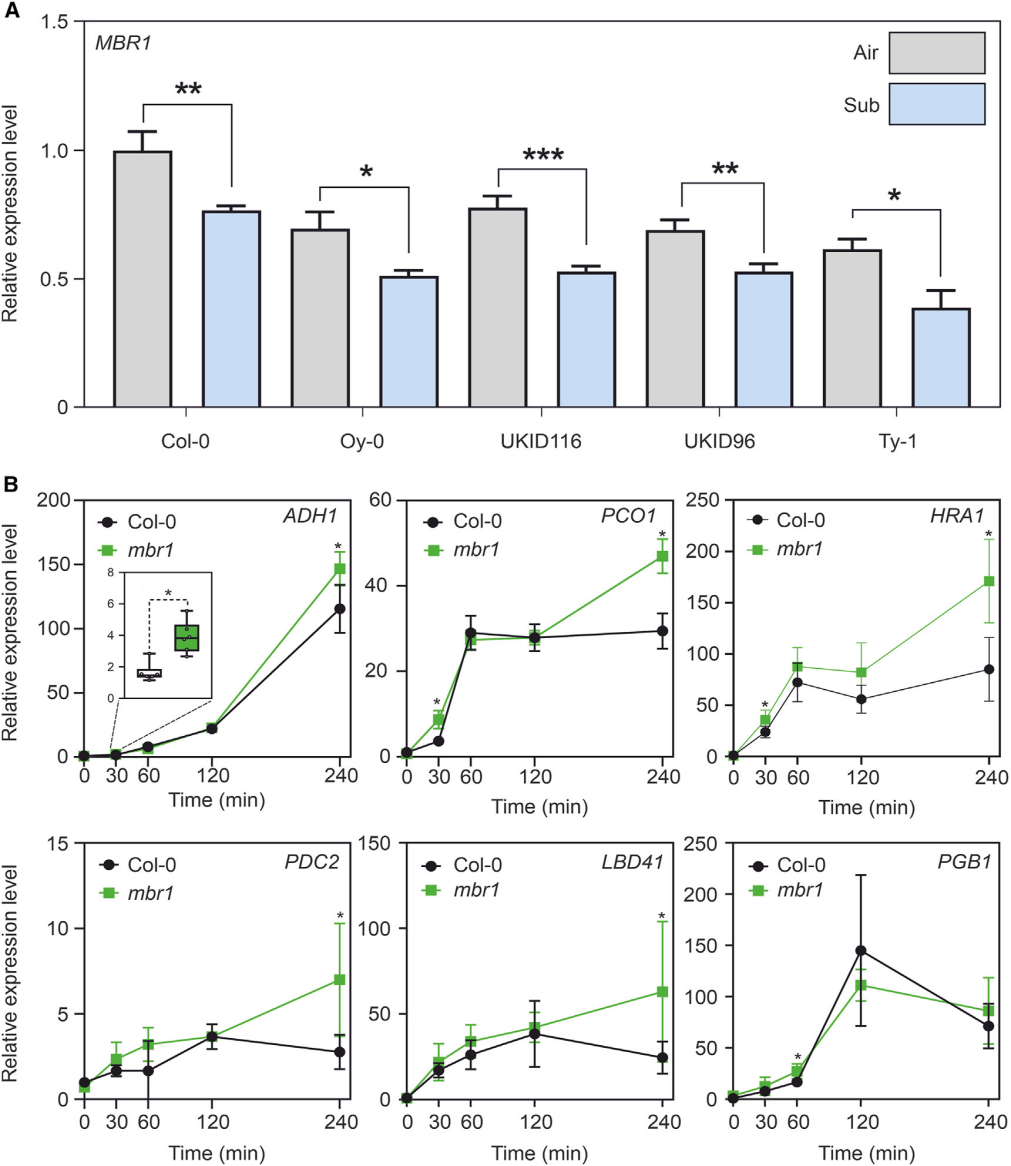
*MBR1* gene expression analysis and impact of the *mbr1* mutation on the expression of selected hypoxia-related genes. **(A)***MBR1* transcript level after 4 h of submergence (Student’s *t* test; ∗*p* < 0.05; ∗∗*p* < 0.01; ∗∗∗*p* < 0.001; ∗∗∗∗*p* < 0.0001). **(B)** Time course of the expression levels of a selection of hypoxia-responsive genes in Col-0 and *mbr1* during submergence; statistically significant differences are indicated by asterisks (Student’s *t* test; ∗*p* < 0.05; ∗∗*p* < 0.01; ∗∗∗*p* < 0.001; ∗∗∗∗*p* < 0.0001).

To further explore the role of *MBR1* in the hypoxic response, we evaluated gene expression levels using RT–qPCR. Expression of *MBR1* was evaluated in Col-0 and the accessions carrying the polymorphism after the plants had been subjected to 4 h of submergence. The results showed significant downregulation of *MBR1* in all analyzed ecotypes (Figure 4A). This repression of *MBR1* during submergence stress might be associated with reduced activity of the protein, which could favor the stability of MED25 to ensure a more robust hypoxic response. We next subjected *mbr1* knockout plants, together with the WT, to submergence for 4 h and collected data during a time course (30 min, 1 h, 2 h, 4 h) to check whether the absence of *MBR1* might play a role during hypoxic stress. Within the initial 30-min period, expression levels of the core hypoxia genes *ADH1*, *PCO1*, *HRA1*, *PDC2*, and *LBD41* were upregulated in *mbr1* compared with the WT (Figure 4B). This result suggests that *MBR1* may normally dampen the hypoxia response in the WT, possibly by affecting the stability of MED25. The gene expression levels were then equalized to those of the WT after the first hour of submergence. This may be because, in the WT, *MBR1* is also downregulated after the first hour of submergence and eventually reaches expression levels similar to those of the *mbr1* mutant. The expression of *PGB1* was only slightly affected in *mbr1*, with upregulation 1 h after submergence (Figure 4B). To further investigate the temporal dynamics of hypoxia-related gene expression, an additional experiment was performed at the 12-h time point. The results revealed no significant differences in gene expression between the experimental and control groups at this later time point (Supplemental Figure 8).

### MED25 is more stable in ecotypes that carry the polymorphism

**Figure 5:**
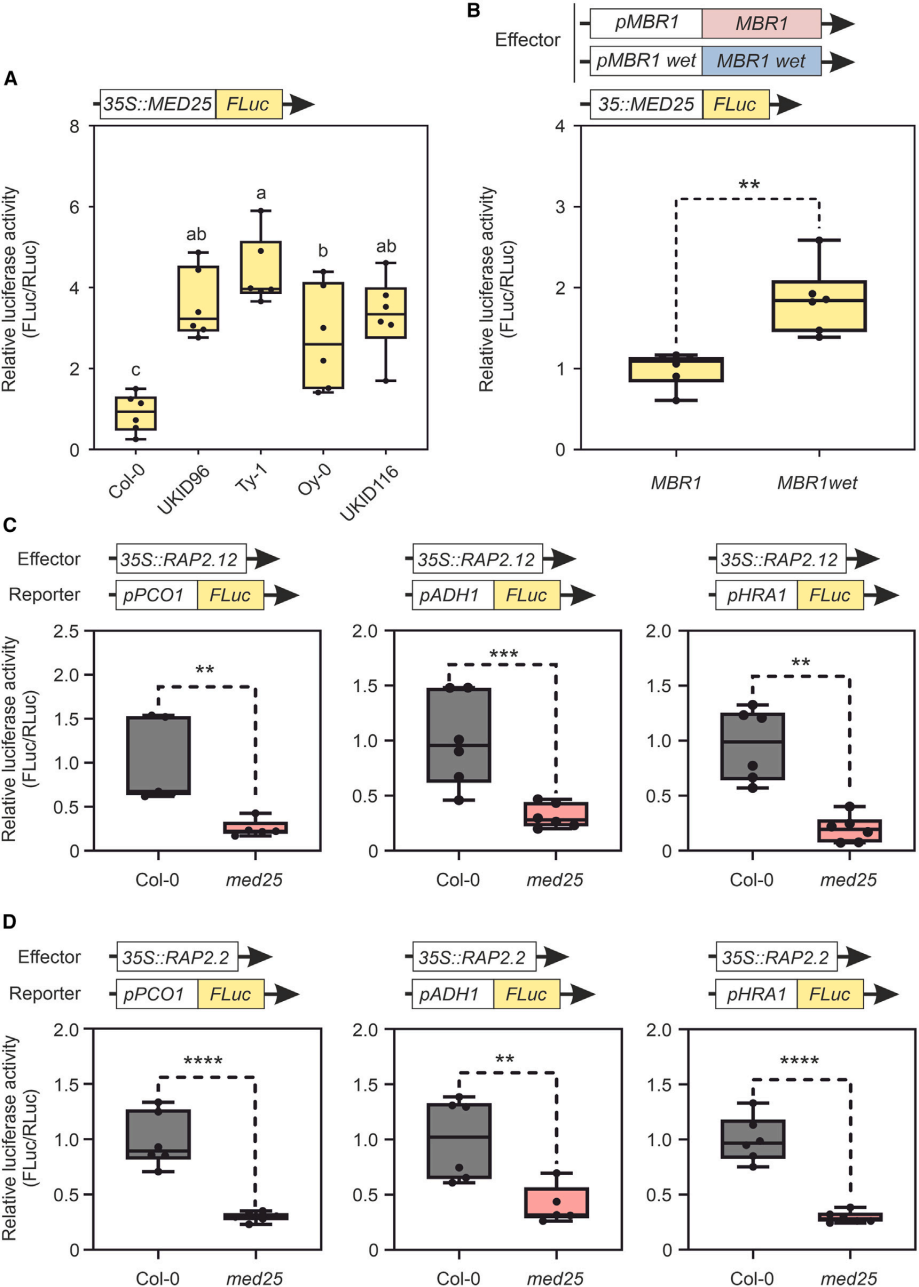
MED25 is required for the induction of hypoxia-responsive genes by RAP2.12 and RAP2.2. **(A)** Relative transcriptional activity of MED25 in Col-0 protoplasts and accessions carrying *MBR1wet*. **(B)** Relative transcriptional activity of MED25 in protoplasts of *mbr1* transformed with two versions of *MBR1*, the WT version (−) and the version with the polymorphisms (+). **(C)** Relative transcriptional activity of the promoters of *PCO1*, *ADH1*, and *HRA1* in protoplasts of Col-0 and med25 transformed with an overexpressor of RAP2.12 after 4 h of hypoxia at 1%. **(D)** Same as **(C)**, but protoplasts were transformed with an overexpressor of RAP2.2. In the boxplots, dots represent single data points, the whiskers denote the minimum/maximum values, the box defines the interquartile range, the center represents the median, and the box borders represent the lower and upper quartiles. Statistically significant differences are indicated by asterisks (Student’s *t* test; ∗*p* < 0.05; ∗∗*p* < 0.01; ∗∗∗*p* < 0.001; ∗∗∗∗*p* < 0.0001). Different letters (a, b, c, ab) indicate differences in ANOVA tests (Tukey’s post hoc test, *p* < 0.05).

To test whether the MBR1 variant influences the stability of MED25, we performed a dual luciferase reporter assay to quantitatively measure the effect of *MBR1* on MED25 stability. Col-0 and ecotypes with *MBR1wet* were transformed with the *35S::MED25::FLuc* construct. Our findings showed that ecotypes with the MBR1 variant exhibited significantly higher relative luciferase activity than Col-0 (Figure 5A).

To determine whether the increased stability of MED25 was due to the *MBR1wet* allele or to background variation, we cloned the WT *MBR1* version from Col-0 and the *MBR1* version containing missense mutations from the Ty-1 ecotype, which, according to the precipitation datasets, was the accession that came from the rainiest environments. The vectors were then used for co-transformation, starting from a knockout *mbr1* genotype to ensure that there was no endogenous form of the protein. Protoplasts isolated from the plants were co-transformed with one of the *MBR1* versions together with the *35S::MED25:FLuc* vector. The results showed that for the *MBR1wet* allele, there was greater MED25 activity, indicating that the stability of MED25 is related to *MBR1* and its alternative forms (Figure 5B).

### MED25 is required for a complete hypoxic response

To confirm the role of MED25 in the establishment of the hypoxic response, we performed luciferase assays with protoplasts of the Col-0 and *med25* knockout genotypes. Promoters of the hypoxia-related genes *ADH1*, *PCO1*, and *HRA1* were cloned and fused with luciferase. These constructs were co-transformed with effectors, represented by overexpressors of the *RAP2.12* and *RAP2.2* genes, into Col-0 and *med25* protoplasts. The results demonstrated significantly reduced relative luciferase activity in all experiments (Figure 5C and 5D). In the absence of *med25*, a full hypoxic response was unattainable, confirming the indispensable role of MED25.

## Discussion

The ability of a species to withstand the challenges of climate change hinges on its genetic diversity (Exposito-Alonso, 2023). Adaptation to shifting environments typically involves the gradual fine-tuning of molecular mechanisms, which occurs over evolutionary timescales. However, the current climate crisis is now fast-tracking these changes, putting pressure on organisms that are likely unable to adapt rapidly enough (Hoffmann and Sgró, 2011). The big data revolution in genomics and remote climate sensing has led to new ways of dealing with these challenges, leveraging the diversity available in natural populations and connecting it to traits with clear adaptive significance. This knowledge could be used for the conservation and sustainable exploitation of biodiversity; however, it requires a clear description of the mechanisms underlying adaptation.

Here, we combine an eGWAS approach with molecular biology to identify and validate genes of adaptive significance whose extant allelic variation is the result of evolutionary processes.

The results of environmental association analyses highlighted strong signs of association between rainfall and one gene, *MBR1*. This gene is known to regulate MED25 stability (Iñigo et al., 2012). MED25 is a subunit of the Mediator multiprotein complex and is involved in a wide range of plant functions (Kazan, 2017). However, its key role is as a coactivator in the regulated transcription of genes dependent on RNA polymerase II. The Mediator complex functions as a bridge to convey information from gene-specific regulatory proteins to the basal RNA polymerase II transcription machinery. The mediator is recruited by promoters to direct interactions with regulatory proteins and serves as a scaffold for the assembly of a functional preinitiation complex with RNA polymerase II and general TFs (Allen and Taatjes, 2015; Dolan and Chapple, 2017). Among the TFs recruited by MED25 are *RAP2.2* and *RAP2.12* (Ou et al., 2011; Shukla et al., 2019), which are two of the central regulators of the hypoxic response.

Recently, Schippers et al. (2024) extensively validated the function of MED25 in hypoxic stress. Their study demonstrated that TFs from the ERF family are recruited by MED25 to enable the full activation of hypoxia response mechanisms. Our findings converge on the same conclusions, identifying MED25 as a crucial regulator of the plant hypoxic response. The absence of MED25 results in significantly diminished induction of the core hypoxia-responsive genes. MED25 interacts with key ERF-VII TFs, facilitating their binding to target gene promoters and thereby orchestrating a comprehensive transcriptional response to low-oxygen conditions. Our data corroborate these insights, showing that without MED25, plants exhibit a notably weaker activation of genes essential for coping with hypoxic stress. This highlights the indispensable role of MED25 in mediating the transcriptional network that underpins the survival and adaptation of the plant during episodes of oxygen deprivation.

The close connection between *MBR1*, MED25 and hypoxia led us to a compelling hypothesis. *MBR1* may have been subjected to natural selection in relation to rainfall, as it plays a role in modulating the hypoxic response, triggering a cascade of events that directly influence the stability of MED25 and thus influencing the activity of ERF-VII TFs.

The influence of natural selection upstream of TFs is conceivable, as mutations within these factors could have detrimental consequences (Mitsis et al., 2020). Indeed, recent research has underscored the notable stability of crucial regulatory factors that govern plant physiological responses, reducing the likelihood of mutations (Monroe et al., 2022). However, one recent study revealed that accessions from distinct environments achieve adaptation through specific *cis*-element modifications, leading to increased constitutive expression of *RAP2.12*. This highlights how evolution can directly impact the regulatory elements involved in oxygen sensing (Lou et al., 2022). This and other studies have shown how specific mutations can contribute to the adaptation and thriving of *Arabidopsis* in particular environments. The study by Guo et al. (2023) revealed that natural variation in the *SVP* (SHORT VEGETATIVE PHASE) allele of the MADS-box gene in *A. thaliana* has a pleiotropic effect on plant adaptation to contrasting environmental conditions. Specifically, they identified a non-functional mutation in the SVP-32V allele that alters normal regulatory interactions with target genes such as *GRF3*, *CYP707A1/3*, and *AtBG1*. This resulted in increased leaf size and greater tolerance to wet conditions but reduced drought tolerance. Similarly, Kang et al. (2023) observed a specific insertion in the promoter region of the HPCA1 gene in the Tibet-0 ecotype, which increases gene expression and promotes adaptation to alpine environments.

The SNPs found within the *MBR1* gene have a low MAF (0.02), and the allele associated with high rainfall is restricted to populations in England and Scandinavia. *Arabidopsis* established relatively recently in northern Europe (Platt et al., 2010; Horton et al., 2012). Considering these findings, we may speculate that there is a correlation between the presence of the allele and the relatively recent northward expansion of *Arabidopsis*, which would be consistent with the species’ adaptive response to challenges posed by submergence. Certainly, this should be interpreted within the framework of a multi-locus process of adaptation, as corroborated by the observed correlation between genetic clusters and precipitation amounts (Supplemental Figure 4).

Although geography and evolutionary history may have influenced the differentiation of these genetic groups, our results strongly imply that adaptive processes are associated with precipitation patterns. With this evidence, we found that *MBR1* was significantly associated with soil bulk density, which bears no correlation with rainfall, further supporting the possible role of this gene in the response to hypoxia during waterlogging.

The close correlation between bulk density and the possibility of soil flooding is interesting. Soils with a high bulk density are typically more compacted and have a limited capacity to absorb and drain water during extreme rainfall events. As a result, water may accumulate on the surface, leading to poor drainage and increasing the risk of waterlogging and therefore the establishment of a hypoxic environment (Manik et al., 2019).

One frequent, significant limitation of the current eGWAS literature is the lack of experimental validation for genes identified through association studies (Lasky et al., 2023). Our aim was to specifically address this limitation by conducting comprehensive experimental investigations to confirm the role of the identified candidate genes. This approach aimed to bridge the gap between genetic associations and functional insights, thereby contributing to a more comprehensive understanding of the mechanisms underpinning plant adaptation.

Through experimental investigations, we successfully revealed the role of *MBR1* in hypoxia stress. We observed that plants from rainy environments displayed superior tolerance to submergence stress. However, because this resistance could potentially be linked to a multigenic effect, we used *MBR1* and *MED25* knockout lines for waterlogging and submergence experiments, revealing that *mbr1* demonstrated better resistance than Col-0 and *med25* under both conditions (Figure 3). This result clarified the potential involvement of the MBR1/MED25 module in the hypoxia response machinery and was further supported by qPCR experiments demonstrating greater hypoxic gene induction in *mbr1* than in Col-0 (Figure 4). Luciferase assays indicated that accessions from rainy environments exhibited greater stability of MED25 due to reduced *MBR1* activity (Figure 5B). Lastly, we provided the first direct evidence of the essential role of MED25 in establishing a comprehensive hypoxic response (Figure 5C and 5D).

Our research is aimed at understanding the intricate ways in which plants adapt to their surroundings. Our results show that existing databases, such as the 1001 Genomes Project, already hold a treasure trove of information waiting to be extracted. We demonstrate that tools such as eGWASs can serve as a compass for unearthing genes closely intertwined with species adaptation to distinct, often harsh environments. The discovery of a mutation in the *MBR1* gene that leads to a cascade effect on hypoxia tolerance offers a fascinating glimpse into the intricate molecular mechanisms underlying plant adaptation to challenging environments.

**Figure 6:**
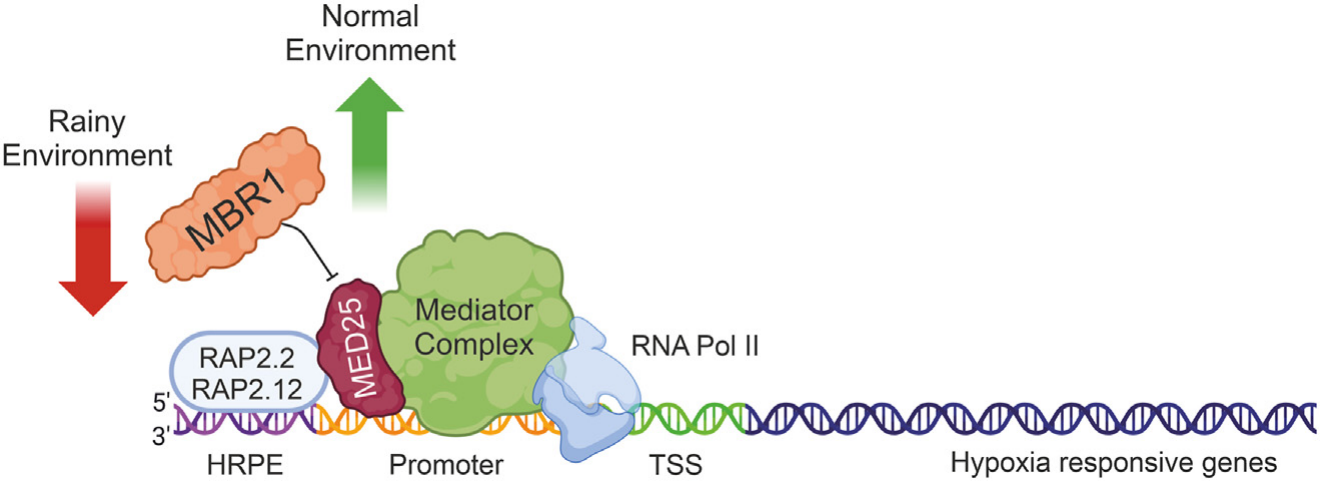
Proposed model based on our findings. Plants that inhabit particularly rainy environments have acquired mutations in the *MBR1* gene, leading to limited activity of MBR1 as a repressor of MED25. This limited activity results in increased MED25 activity and, consequently, more effective activation of the ERF-VII-dependent (RAP2.2; RAP2.12) hypoxic response in the plant.

Exploring how organisms perceive oxygen and respond to extreme environments (Abbas et al., 2022) not only enriches our knowledge but also charts promising pathways for understanding adaptive strategies amid evolving climate challenges. In addition to revealing a novel function of the *MBR1* gene, we have ventured into uncharted territory by revealing the role of MED25 in the hypoxic response. Notably, when this protein is inactive, there is a stark reduction in the activity of core anaerobic genes, underscoring the indispensability of MED25 for the full functionality of ERF-VII TFs (Figure 6).

In conclusion, our study aims to serve as a bridge connecting two domains of genetics: forward and reverse genetics, as well as an attempt to unite the potential of emerging tools, such as eGWASs, with cutting-edge molecular techniques. Although these two approaches provide valuable information independently, the divide between them often leaves crucial gaps in our understanding of genetic mechanisms. By forging this connection, we aimed to paint a more comprehensive picture of adaptation processes.

## Methods

### Plant materials and diversity analysis

The *Arabidopsis* accessions used in this study are part of the 1001 Genomes Project (Alonso-Blanco et al., 2016), which consists of 1135 natural inbred *A. thaliana* ecotypes (or accessions) with their respective DNA sequencing data. Starting with the overall dataset, accessions were filtered out to retain the most informative set of ecotypes. Pairwise genome-wide identity-by-state differences were calculated using PLINK version 1.9 (Purcell et al., 2007). When the pairs differed by <0.01 changes per polymorphic site, we randomly removed one member of the pair. The VCF file with SNP calls from the 1001 Genomes Project (https://1001genomes.org/data/GMI-MPI/releases/v3.1/) was therefore filtered with BCFtools version 1.12 (Li, 2011), resulting in the retention of 934 ecotypes (Supplemental Table 4).

The population structure and genetic clusters for the 934 *Arabidopsis* accessions were reconstructed using ADMIXTURE version 1.3.0 (Alexander et al., 2009). ADMIXTURE is a clustering software that infers population ancestries starting from highly informative, unlinked SNP data. The starting VCF file was restricted to biallelic SNPs with a genotype calling rate >95% and a minimum allele frequency >1% using BCFtools. The dataset was then LD pruned using PLINK. ADMIXTURE was then run with a 20-fold cross-validation (K = 2 to K = 20). The resulting best value of K, represented by the lowest cross-validation error value, was selected as the number of resulting clusters.

### Precipitation data, soil data, and correlation analysis

Geospatial analyses were performed with the Geographic Information System software QGIS version 3.16 (QGIS Association, 2022). The Global Positioning System coordinates for each of the 934 accessions were derived from the 1001 Genomes Project database. The corresponding climatic data were retrieved from WC (Fick and Hijmans, 2017). Rainfall measures were also derived from the climatologies at high resolution for the Earth’s land surface areas (CHELSA) (Brun et al., 2022), the Climate Research Unit (Harris et al., 2020), and the Global Precipitation Climatology Center (Schneider et al., 2011). Association analyses for each individual month were performed using the WC historical monthly weather dataset.

Considering the close correlation between hypoxic stress and soil water retention capacity, we extrapolated soil physical attributes from the SoilGrids250m version 2.0 database of the International Soil Reference and Information Centre (Hengl et al., 2017). Information on the bulk density, clay content, silt content, sand content, and coarse fragments was collected. Full information on the pedoclimatic variables used in this study can be found in Supplemental Table 5.

Statistical analyses were performed with custom scripts in R (R Development Core Team, 2020), unless stated otherwise. Precipitation and soil variables were checked for correlation using Pearson’s test (*r*). A PCA was performed to reduce the complexity and dimensionality of the rainfall dataset. To reveal the influence of precipitation patterns on the potential divergence of genetic clusters, we integrated precipitation data with the ADMIXTURE-based membership probabilities of each genome. Statistical analyses (Student’s *t* test) were performed using the R/*ggpubr* package, and plots were produced using R/*ggplot2* (Wickham, 2016).

#### eGWAS and LD analysis

The eGWAS was performed using an Efficient Mixed-Model Association eXpedited (EMMAX) (Kang et al., 2010) algorithm as implemented in the web-based application easyGWAS (Grimm et al., 2017). EMMAX performs statistical tests for association mapping while also accounting for population structure. EMMAX fits a model as: $Y=\mu +X_{i}\beta +Zu+\varepsilon$, where Y is the vector of trait values, μ is the mean value, X is the alternative allele dosage at SNP i, and β is the allelic effect of SNP i on the trait. The structure is corrected with a random genotype term represented by u, which follows a multivariate normal distribution $N\left(0,A\sigma_{G}^{2}\right)$, where A is the relationship matrix between all individual genotypes built from SNP information, and $\sigma_{G}^{2}$ is the genotype-associated variance. SNPs were considered to be significantly associated with a pedoclimatic variable when they exceeded the Bonferroni threshold for a nominal test with α=0.05. To further reduce the possibility of false positives, we focused on genetic variants that showed the strongest statistical association (top hits). Quantile-quantile (q-q) plots were used to assess the normality of the dataset and to identify any deviations from the expected distribution. The q-q plots were generated by plotting the quantiles of our sample data against the quantiles of a theoretical normal distribution (Figure 1E; Supplemental Figures 9 and 10). Manhattan plots and q-q plots showing the results of association analyses were produced using R/*CMplot* (Yin et al., 2021). LD analysis was performed using PLINK version 1.9 (Purcell et al., 2007). LD analysis was performed on the genomic region containing highly significant SNPs located on chromosome 2 around the *MBR1* gene. The focus was confined to this specific region to ensure the identification and characterization of alleles that may be in strong LD with the significant SNPs. The LD plot was generated using Haploview version 4.2 (Barrett et al., 2005).

#### Plant material and growth conditions

Associations were experimentally validated using five accessions: Columbia (Col-0), which served as the WT reference genotype for all experiments, together with UKID96, UKID116, Ty-1, and Oy-0, which are natural inbred ecotypes from the 1001 Genomes Project. Inbred ecotypes were selected because they were sampled in regions with the highest rainfall. tDNA insertion mutants for the *mbr1* and *med25* genes were also used. All seeds were obtained from the Nottingham *Arabidopsis* Stock Centre. Seeds were sown in a soil mixture of 70% professional potting medium and 30% perlite, then vernalized at 4°C in the dark for 48 h. They were subsequently germinated at 22°C day/18°C night, with a 12-h photoperiod and 120 μmol photons m^−2^ s^−1^. The plants were grown in pots for 3–4 weeks before being used for the experiments.

#### Submergence, waterlogging, and phenotyping

To test plant responses to flooding and subsequent recovery from stress, plants were submerged (ZT6) in plastic tanks with the water level 10 cm above the leaf level and kept in the dark. For the comparison between the accessions and the Col-0 genotype, plants were subjected to 72 h of submergence. The plants were then removed from the boxes, and the photoperiodic conditions were restored (22°C day/18°C night with a 12-h photoperiod). Recovery from stress was evaluated 1 week after the treatment ended. For the submergence experiment comparing Col-0, *mbr1*, and *med25* knockout genotypes, the plants were subjected to 48-h submergence; plants were then removed from the boxes, and the photoperiodic conditions were restored. Recovery from stress was evaluated 48 h after the treatment ended. For waterlogging treatments, the root systems of 3-week-old plants were immersed in water, but the leaves and petioles were left above the water level.

Before phenotyping, plants were randomized to minimize potential bias and improve the reliability of our results by ensuring that variations in environmental conditions were evenly distributed. Stress resistance was then assessed under waterlogged conditions 20 days after the initiation of waterlogging. Resistance to stress was evaluated as the ratio of the leaf area of control plants grown in air to that of plants that had undergone submergence treatment, i.e., the PLA ratio.

The plants were phenotyped using a LabScanalyzer digital phenotyping machine (LemnaTec GmbH, Aachen, Germany) equipped with a Manta G-1236 camera and a Kowa LM12XC lens. The plant trays were illuminated by two cool white light-emitting diode panels mounted beside the camera at an angle of 30° to prevent direct reflection from the imaging area. The raw images were demosaiced using the Adaptive Homogeneity-Directed Demosaicing algorithm from the OpenCV library and stored as 8-bit PNG images (Ventura et al., 2020).

### RNA extraction and qPCR

Total RNA was extracted as described previously by Perata et al. (1997) with a minor modification (omission of aurintricarboxylic acid) to make the protocol compatible with subsequent PCR procedures. Electrophoresis using 1% agarose gel was performed for all RNA samples to check for RNA integrity, followed by spectrophotometric quantification. Reverse transcription was performed using the Maxima First Strand cDNA synthesis kit for RT–qPCR with dsDNase (Thermo Fisher Scientific). RT–qPCR was performed using 30 ng cDNA and iQ SYBR Green Supermix (Bio-Rad Laboratories) according to the manufacturer’s instructions. *ACTIN2* expression was used as the endogenous control for all genotypes analyzed. A full list of primers used for qPCR is provided in Supplemental Table 6.

#### Construct preparation

To prepare the *35:MED25:FLuc* construct, the coding sequence (CDS) of the gene was amplified from cDNA of Col-0 plants and cloned into the pENTR/D-TOPO vector. The resulting entry vector was recombined into the plasmid *p2GW7L* using Gateway LR Clonase II (Thermo Fisher Scientific). To generate the *pMBR1:MBR1* and *pMBR1wet:MBR1wet* constructs, the promoter and CDS of the gene were amplified from the Col-0 and Ty-1 genotypes, respectively. The *wet* nomenclature is used for the version of the *MBR1* gene carried by accessions that harbor SNPs within the *MBR1* gene.

After recombination with the CDS of *MBR1* or *MBR1wet*, the *p2GW7* plasmid was digested with *SacI* and *SpeI* to remove the CamV 35S promoter. The promoter of *MBR1* or *MBR1wet* was then ligated into the vector using a T4 Anza ligation mix (Thermo Fisher Scientific). To fuse the promoters of *PCO1*, *HYPOXIA RESPONSE ATTENUATOR1* (*HRA1*), and *ALCOHOL DEHYDROGENASE 1* (*ADH1*) to firefly luciferase, the promoters of the respective genes were amplified from DNA extracted from WT plants (Col-0). The isolated promoters were then cloned into pENTR/D-TOPO and recombined in the destination vector *pGW7L*. Lastly, to produce overexpressors of *RAP2.2* and *RAP2.12*, the CDSs of the genes were amplified from WT (Col-0) cDNA, cloned into pENTR/D-TOPO, and recombined into the destination vector. The list of all vectors used in this study is provided in Supplemental Table 7.

#### Isolation and transformation

Protoplasts were isolated from leaves of 3-week-old plants by incubation in enzyme solution (1% w/v cellulase, 0.3% w/v macerozyme, 0.4 M mannitol, 20 mM KCl, 10 mM CaCl_2_, 20 mM MES [2-(*N-morpholino)ethanesulfonic acid] [*pH 5.7]) for 3 h in the dark at 22°C. Protoplasts were then filtered, washed twice with W5 solution (154 mM NaCl, 125 mM CaCl_2_, 5 mM KCl, 2 mM MES [pH 5.7]), and centrifuged for 2 min at 100 × *g* before being resuspended in 0.4 M mannitol, 15 mM MgCl_2_, and 4 mM MES (pH 5.7) to a final concentration of 5 × 10^5^ cells mL^−1^. For transformation, 4 μg of each plasmid was added to 100 μL protoplast suspension, which was then gently mixed with an equal volume of a 40% PEG 4000 solution (0.2 M mannitol, 100 mM CaCl_2_). The mixture was incubated for 20 min at room temperature in the dark, and 440 μL of W5 solution was then added to stop the transformation. The protoplasts were centrifuged at 100 × *g* for 2 min, resuspended in 1 mL of 12 WI solution (50 mM mannitol, 4 mM MES [pH 5.7], 20 mM KCl, 50 mM glucose), and transferred to six multi-well plates. The next day, protoplasts were pelleted by centrifugation for 3 min at 5000 × *g* and flash-frozen in liquid nitrogen for storage.

#### Quantification of luciferase activity

The dual luciferase reporter assay system (Promega) was used to quantify the activities of firefly (*Photinus pyralis*) and *Renilla reniformis* luciferase according to the manufacturer’s instructions. In the case of protoplast transient transformation, firefly luciferase was normalized to *Renilla* luciferase activity using the Lumat LB 9507 tube Luminometer (Berthold).

## Funding

This work was supported by 10.13039/501100007176Scuola Superiore Sant'Anna and by MUR-PRIN2022 (PRIN 2022-2022YHWH9R; Next Generation EU) to P.P. and E.L. This study was carried out within the Agritech National Research Center and received funding from the European Union Next-Generation EU (PIANO NAZIONALE DI RIPRESA E RESILIENZA (PNRR) – MISSIONE 4 COMPONENTE 2, INVESTIMENTO 1.4 – D.D. 1032 17/06/2022, CN00000022).

## Acknowledgments

No conflict of interest declared.

## Author contributions

Conceptualization: S.C., P.P., E.L., and M.D. Methodology: S.C., P.P., E.L., and M.D. Investigation: S.C. and P.M.T. Supervision: P.P., E.L., and M.D. Writing – original draft: S.C. Writing – review & editing: P.P., E.L., and M.D.

## Supplemental information

Supplemental Data 1. Supplemental Tables 1–7

Document S2. Article plus supplemental information
