# Supplementary material for: Environmental genome-wide association studies across precipitation regimes reveal that the E3 ubiquitin ligase MBR1 regulates plant adaptation to rainy environments
Source: Plant Commun. 2024 Aug 31;5(12):101074. doi: 10.1016/j.xplc.2024.101074 (PMC11671751; doi:10.1016/j.xplc.2024.101074)
Supplement: Document S1. Supplemental Figures 1–11 [file mmc1.pdf]

Plant Communications, Volume *5*

Supplemental information

Environmental genome-wide association studies across precipitation regimes reveal that the E3 ubiquitin ligase MBR1 regulates plant adaptation to rainy environments

Simone Castellana, Paolo Maria Triozzi, Matteo Dell'Acqua, Elena Loreti, and Pierdomenico Perata

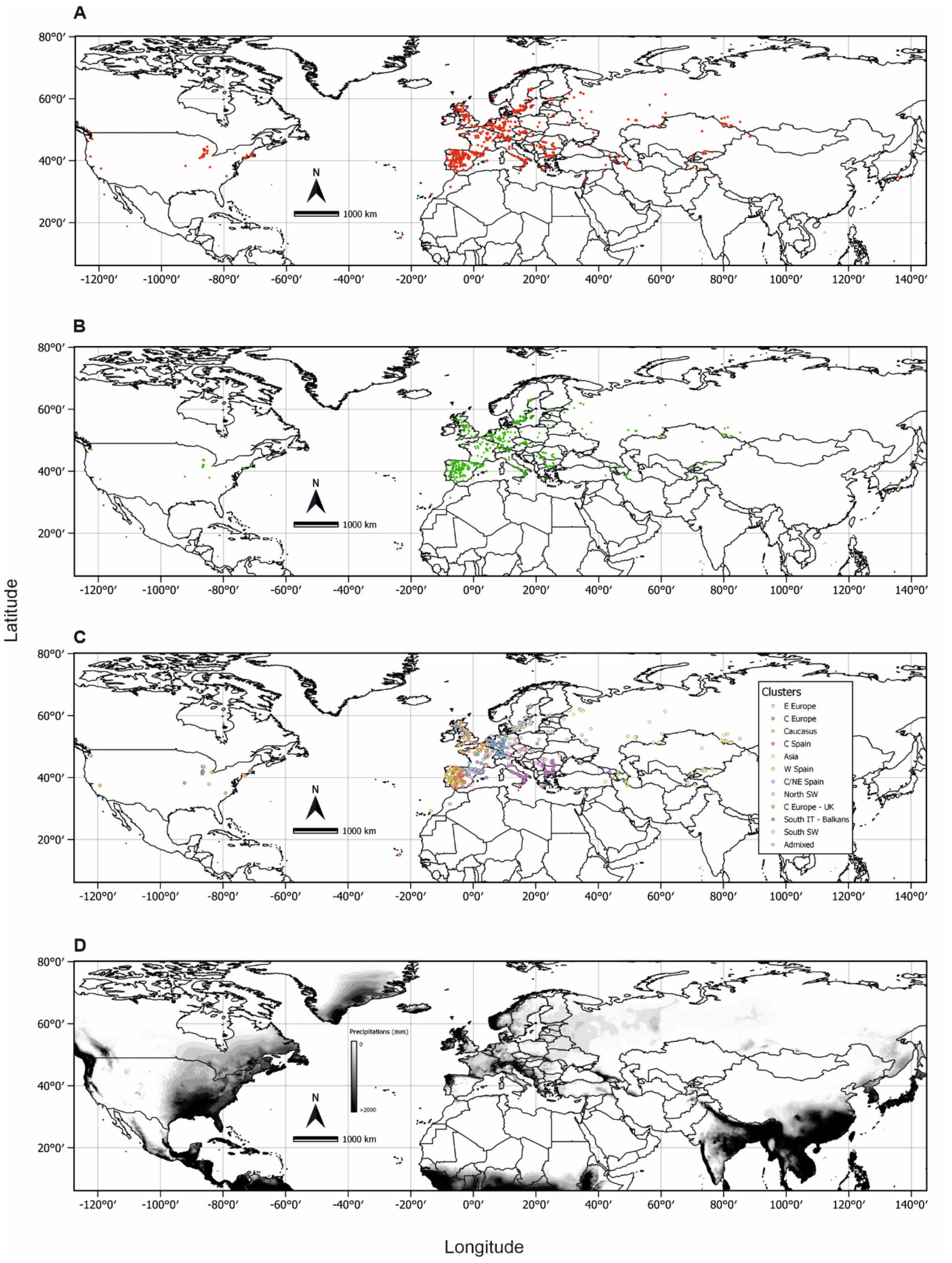

Figure S1. Geographical distribution of the accessions used in the study. Maps representing: **(A)** the distribution of the complete dataset of the 1,335 accessions, **(B)** the distribution of the 934 accessions used for subsequent association analyses after filtering for IBS, **(C)** the distribution of the 11 clusters highlighted by the ADMIXTURE analyses, **(D)** the average annual rainfall for the period 1970 - 2020; the grey scale indicates the average rainfall expressed in millimetres of rainfall.

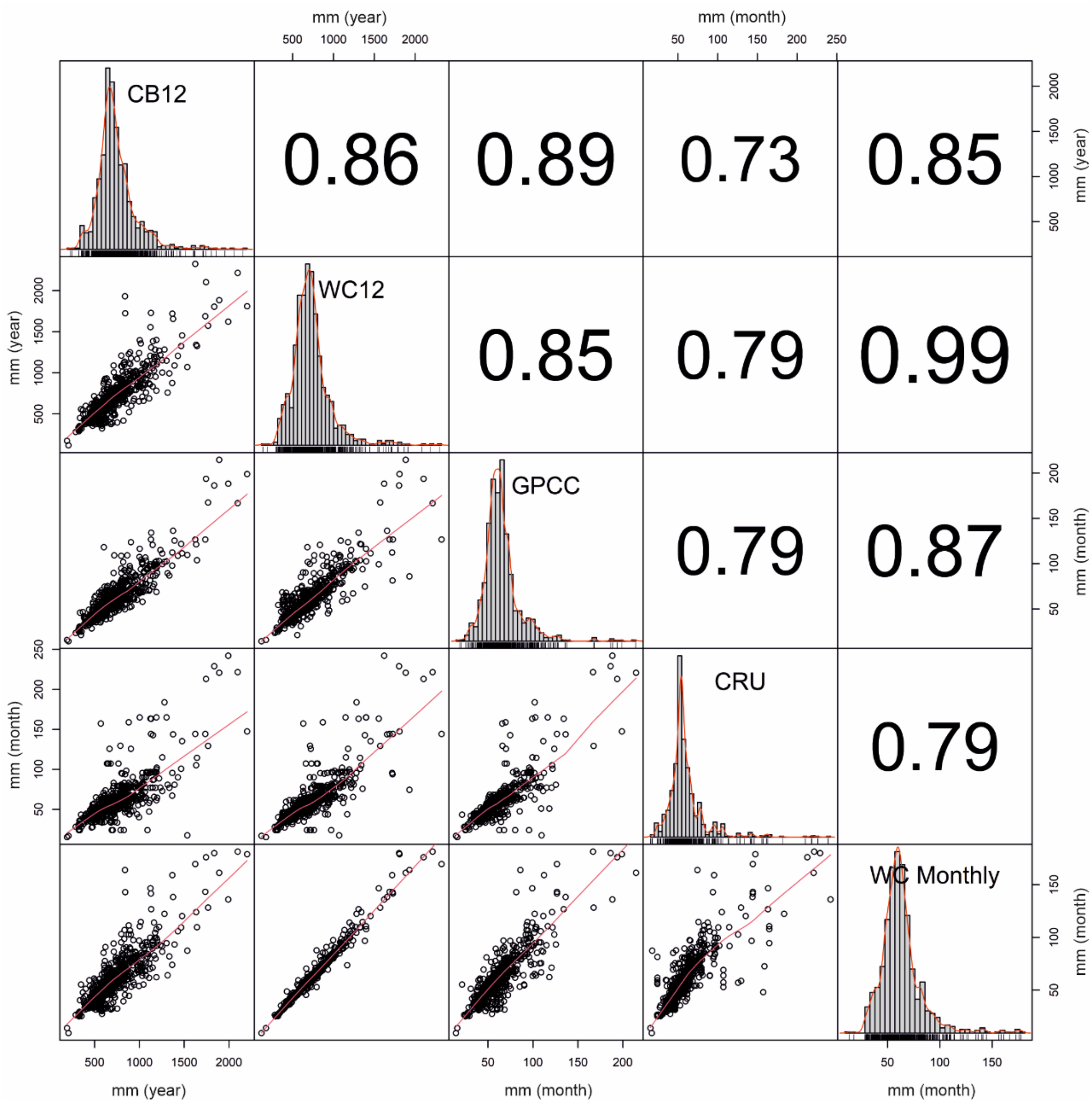

Figure S2. Correlation plot of the precipitation variables used in the study. Pearson's correlation test (*r*) was used.

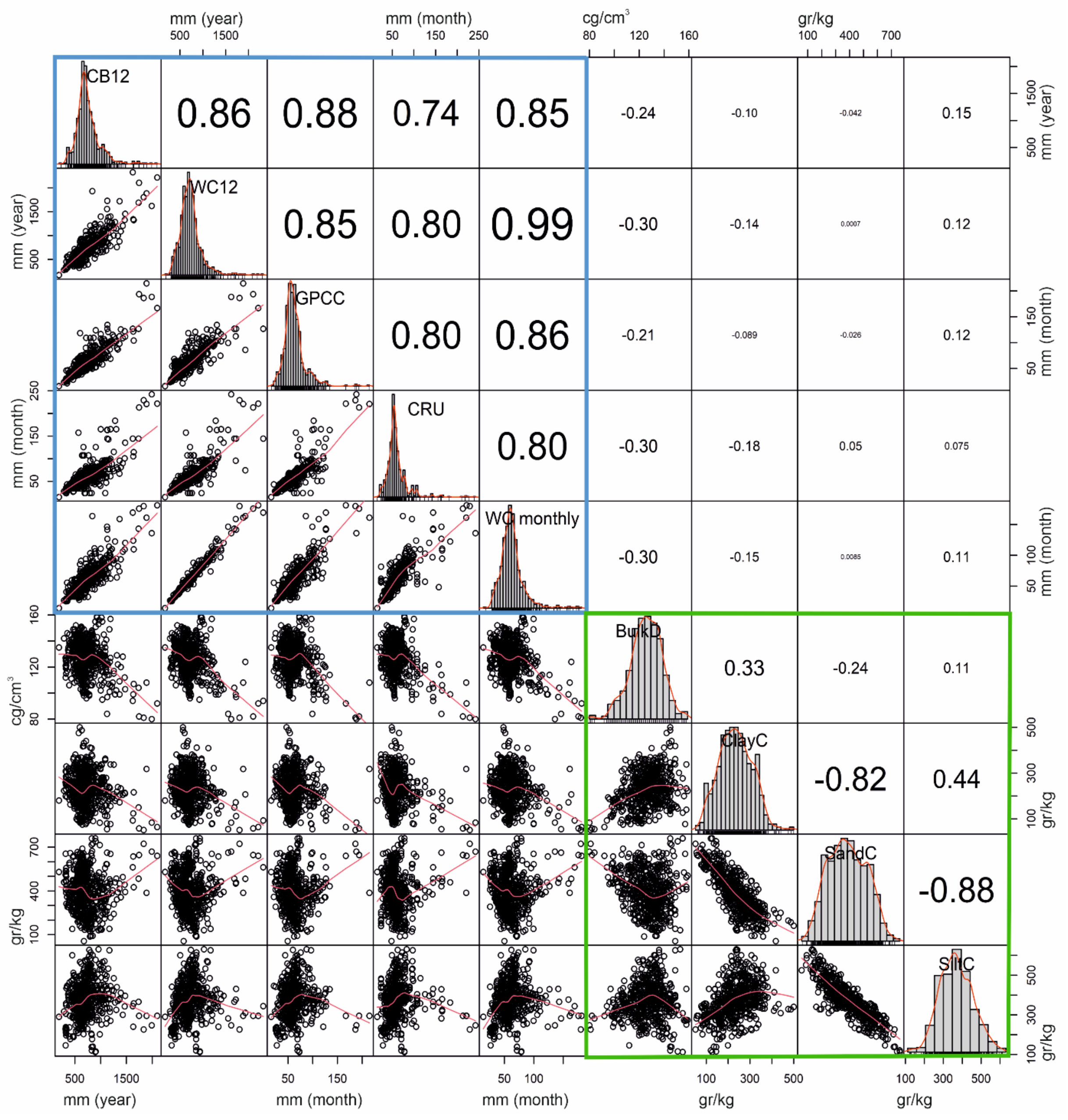

Figure S3. Correlation matrix for the precipitation and soil variables. The upper triangle contains Pearson correlation coefficients (*r*) between each pair of variables; the lower triangle displays scatter plots for the pairs. Diagonal elements show histograms representing the distribution of each variable. Precipitation variables are highlighted within the blue rectangle, while soil variables are highlighted within the green rectangle.

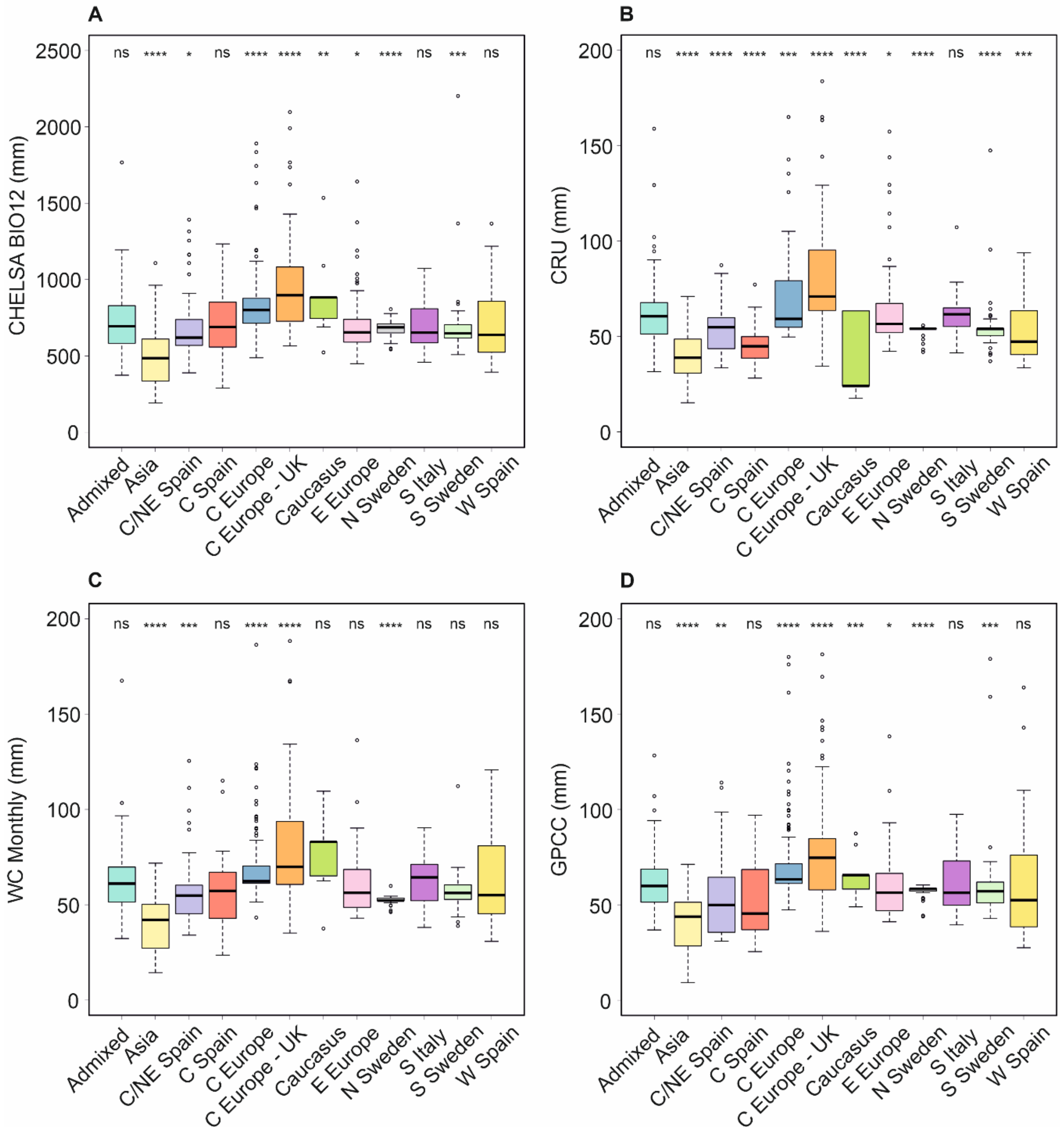

Figure S4. Distribution of the ecotypes based on the precipitation variables used in this study. Different datasets have been used for the analysis: **(A)** CHELSA BIO12 **(B)** CRU **(C)** WorldClim Monthly and **(D)** GPCC. The ecotypes are divided into the 11 clusters highlighted by the ADMIXTURE analyses; statistically significant differences are indicated by asterisks (Student's t-test; ns = non-significant; * = $p < 0.05$; ** = $p < 0.01$; *** = $p < 0.001$; **** = $p < 0.0001$)

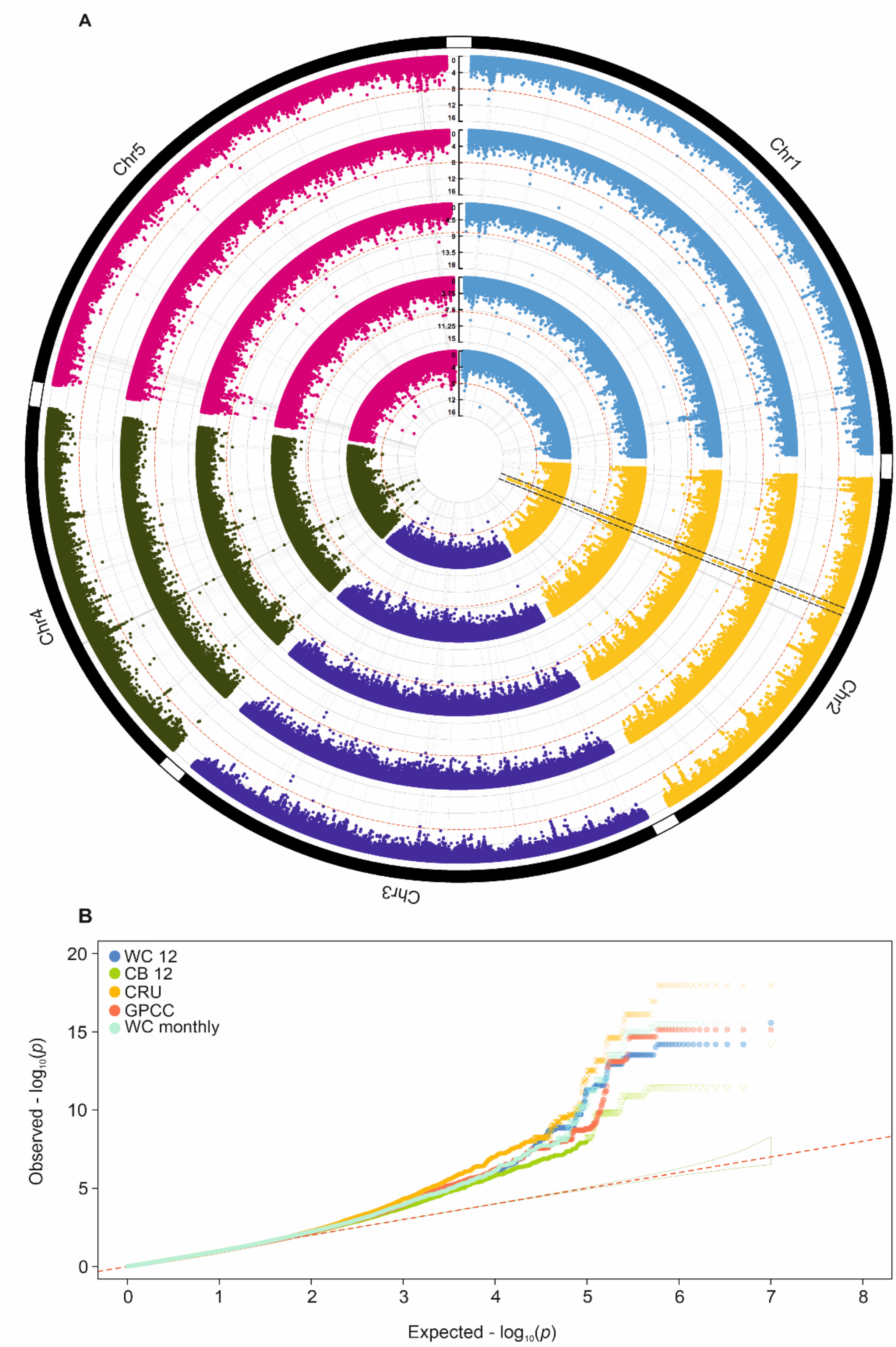

Figure S5. eGWAS for the rain variables. (A) Circular Manhattan plot for the eGWAS analyses of the rain variables. From outer to inner circle: WorldClim BIO 12; CHELSA BIO12; CRU; GPCC and WorldClim monthly precipitations; dashed lines indicate the position of the *MBR1* gene. **(B)** Multitrack Q-Q plot of the eGWAS analysis for the precipitation variables. The region outlined in light blue depicts the 95% confidence interval under the null hypothesis of a uniform *P* value distribution.

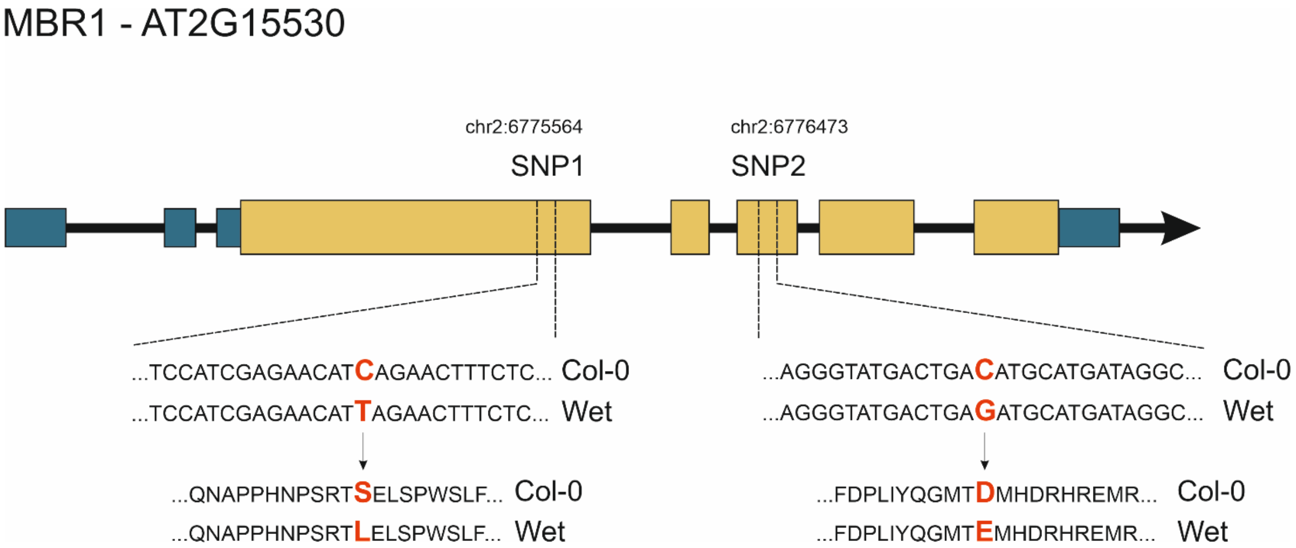

Figure S6. Gene model for *MBR1* depicting the SNPs identified by the eGWAS. The figure highlights the exonic regions where the two polymorphisms identified through environmental association analyses are located, along with their respective positions within chromosome 2. The nucleotide sequences for both the WT and the variant Wet versions are shown, with the corresponding translation into amino acid sequences, illustrating the substitutions resulting from the nonsynonymous nature of the SNPs.

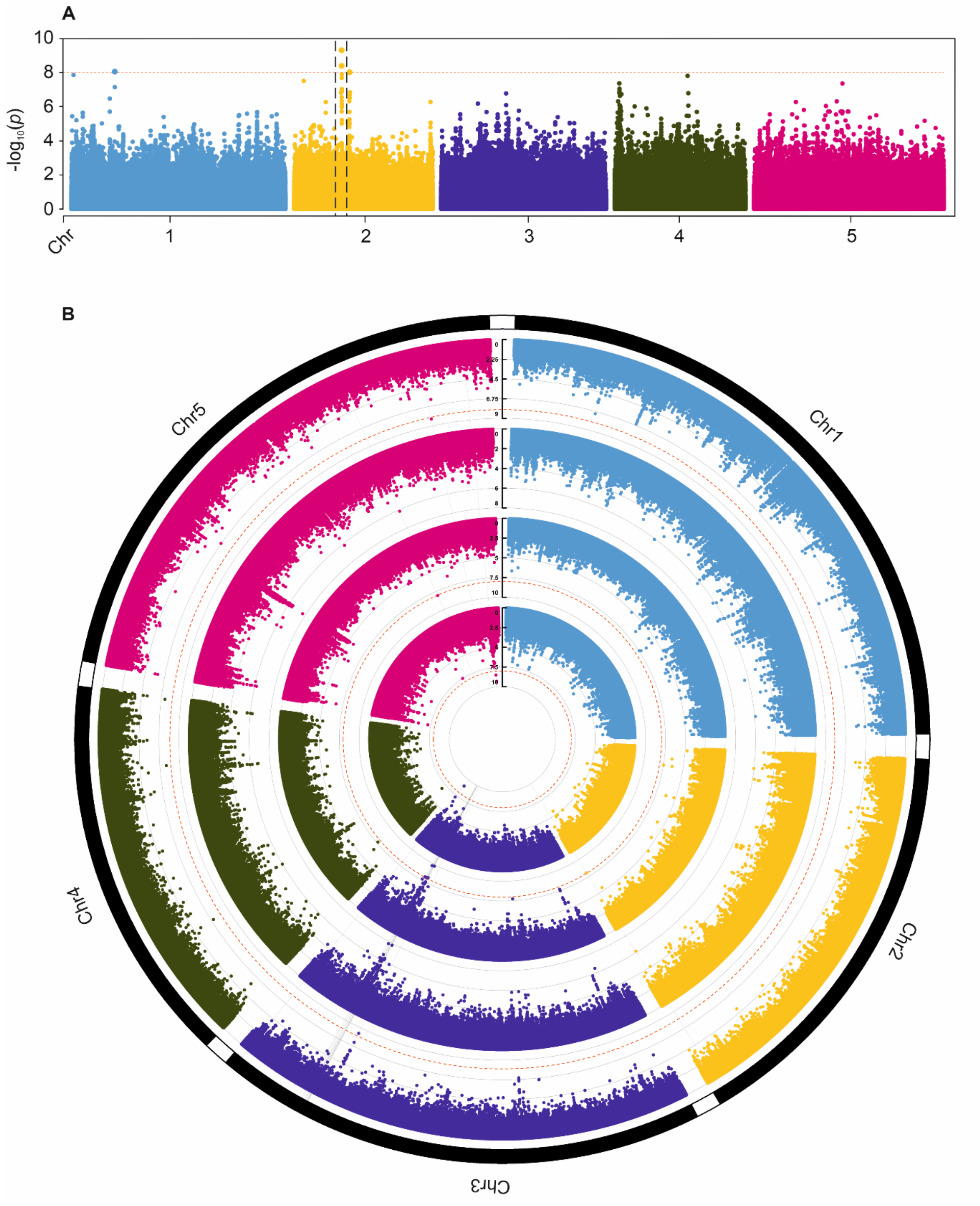

Figure S7. eGWAS for the soil variables. (A) Manhattan plot for bulk density; the dashed lines indicate the position of the *MBR1* gene. **(B)** Circular Manhattan plot for the pedological variables used in the study. From outer to inner circle: clay content; sand content; silt content; coarse fragments

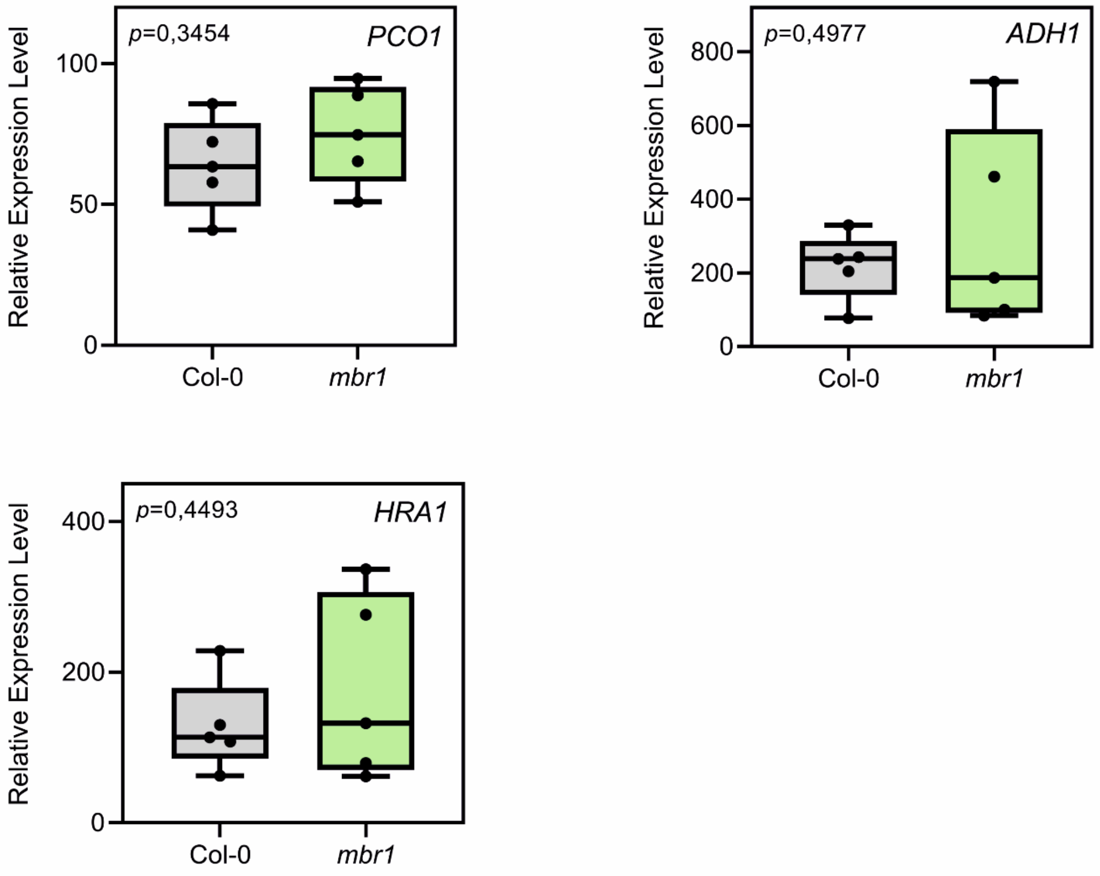

Figure S8. Expression levels of hypoxia-responsive genes (*PCO1*, *ADH1*, and *HRA1*) in Col-0 and *mbr1* genotypes at the 12-hour time point. Three-weeks old plants were submerged in the dark for 12h, while the control plants were kept in air (in the dark). Data are mean ± SD (n=5) and are reported as relative to the aerobic control (set to 1). The p-values (*p*) are shown

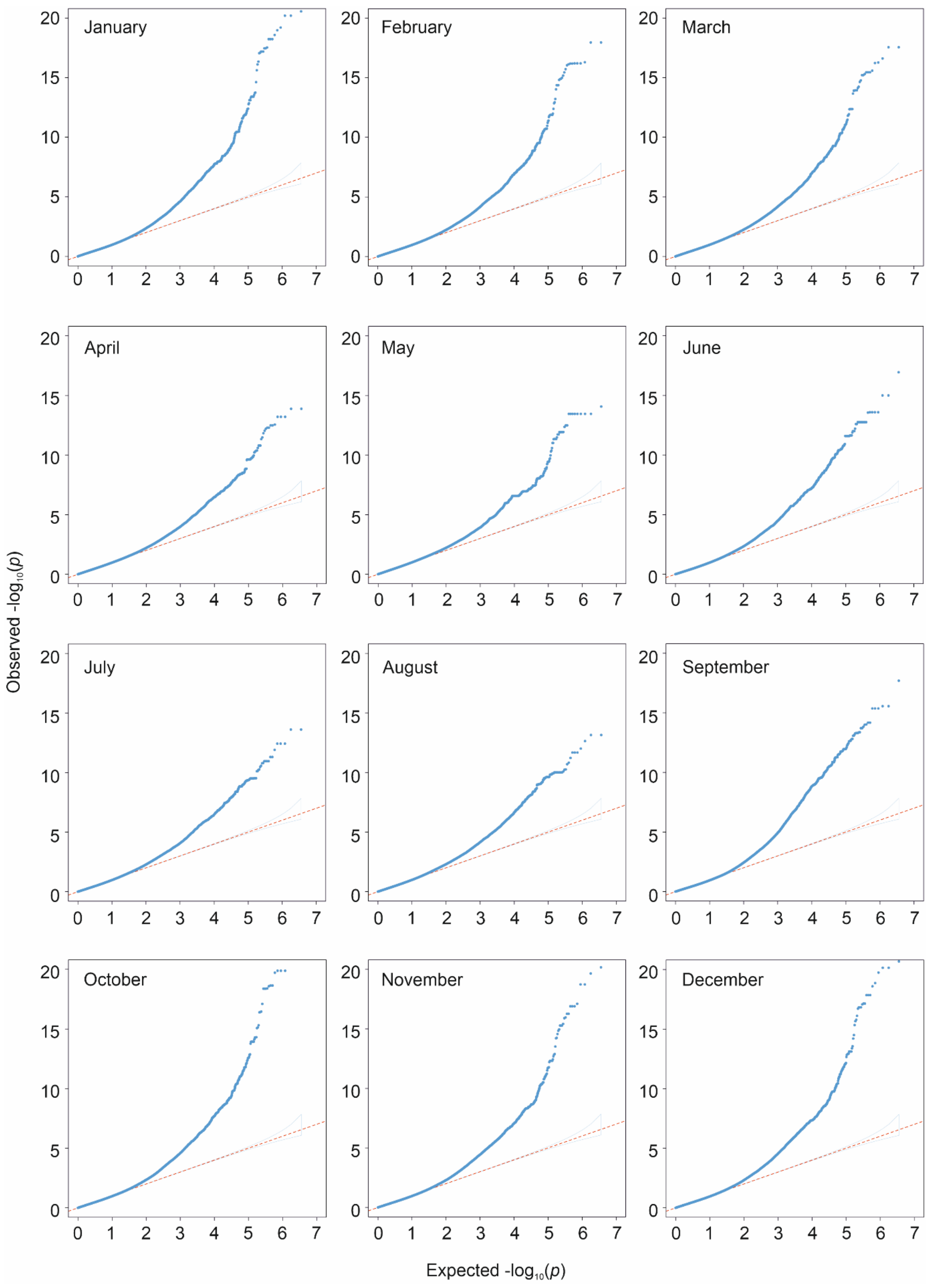

Figure S9. Q-Q plots of eGWAS for the monthly average precipitation. Data for the period 1901 – 2020; the regions outlined in light blue depict the 95% confidence interval under the null hypothesis of a uniform *P* value distribution.

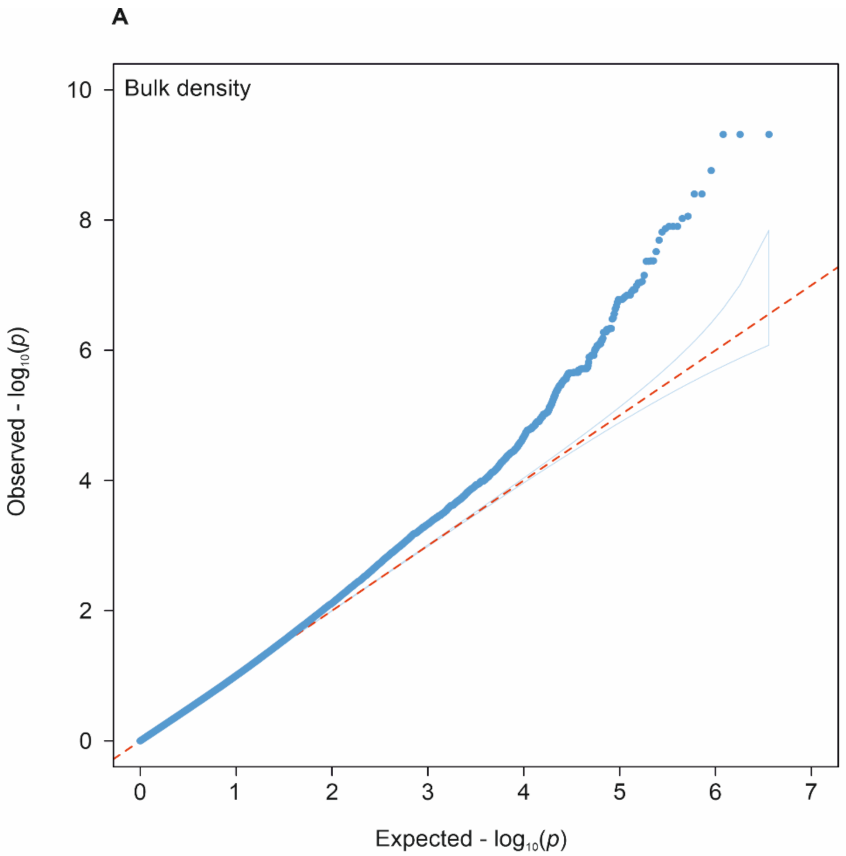

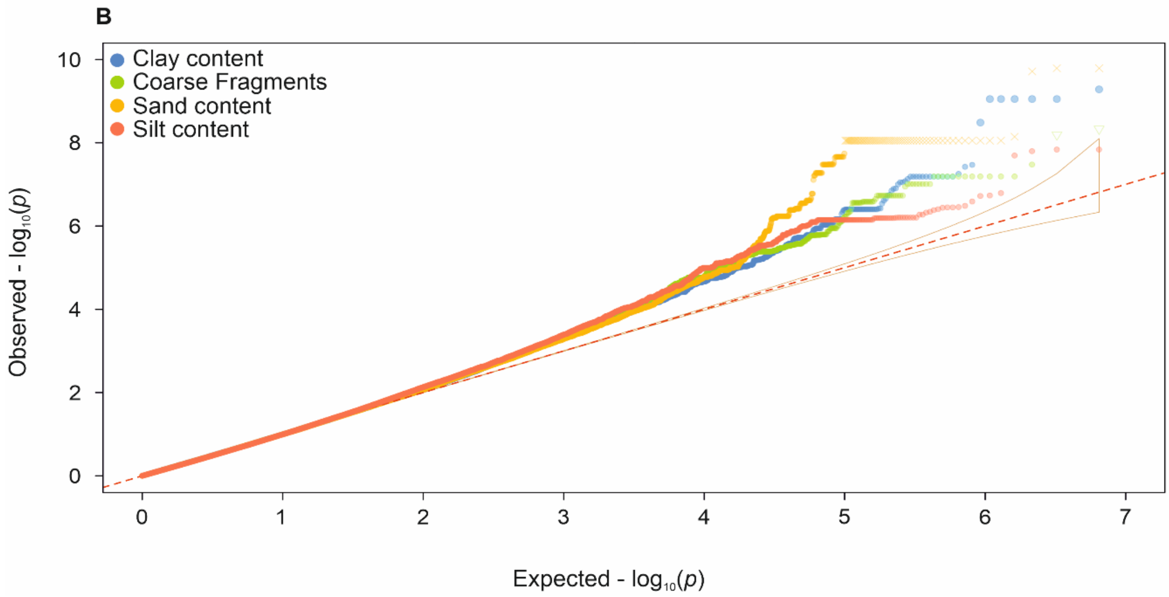

Figure S10. Q-Q plot for the soil variables. (A) Q-Q plots of eGWAS for bulk density. **(B)** Q-Q plots of eGWAS for other soil variables used in the study; the regions outlined in light blue and light red depict the 95% confidence interval under the null hypothesis of a uniform *P* value distribution.

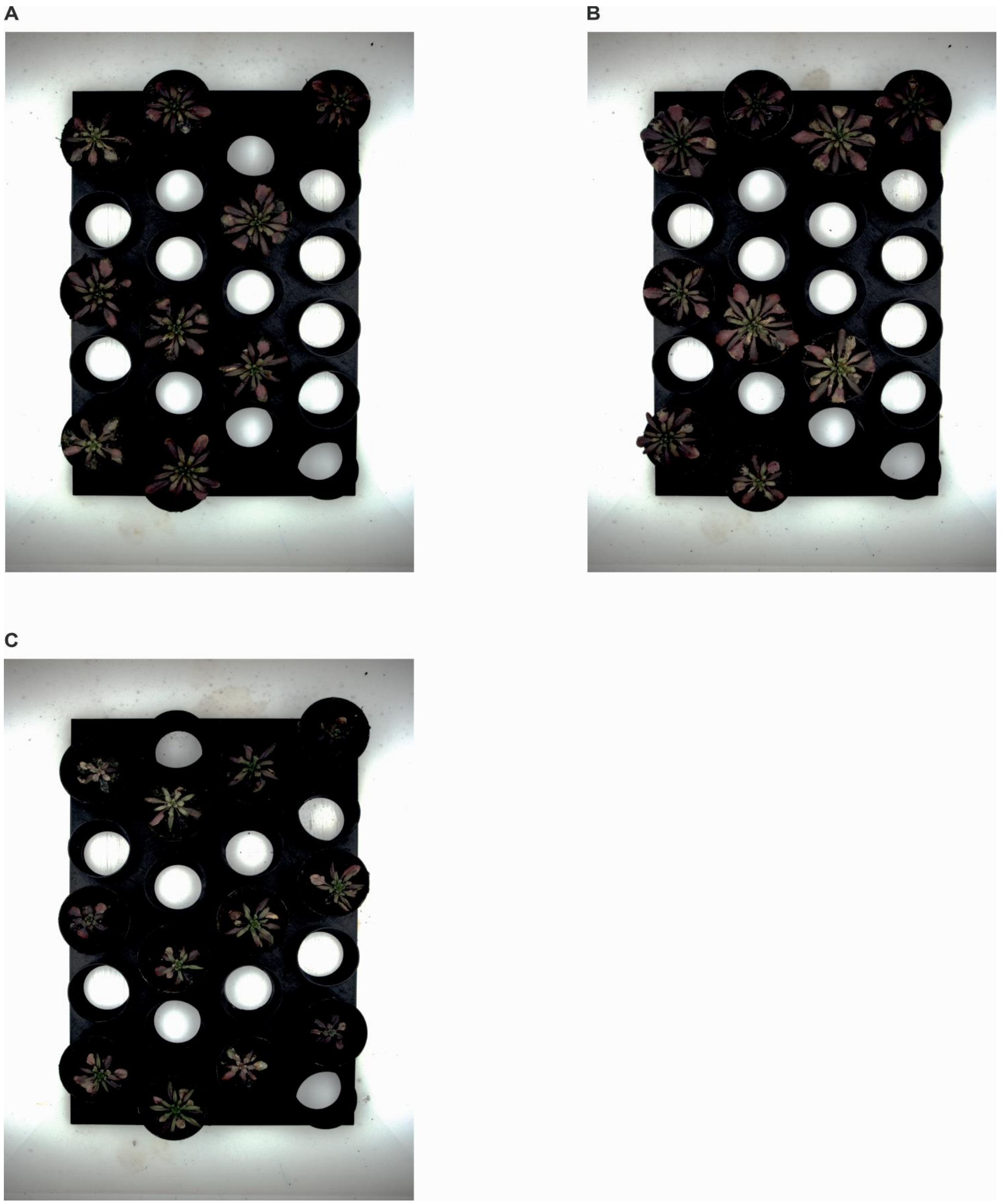

Figure S11. Unedited images sourced for Figure 2b. (**A)** Col-0 (**B)** *mbr1* (**C)** *med25*
